# Identification of the principal neuropeptide MIP and its action pathway in larval settlement of the echiuran worm *Urechis unicinctus*

**DOI:** 10.1186/s12864-024-10228-y

**Published:** 2024-04-03

**Authors:** Zhi Yang, Long Zhang, Wenqing Zhang, Xinhua Tian, Wenyuan Lai, Dawei Lin, Yuxin Feng, Wenwen Jiang, Zhengrui Zhang, Zhifeng Zhang

**Affiliations:** 1 Key Laboratory of Tropical Aquatic Germplasm of Hainan Province, Sanya Ocean Institute, Ocean University of China, Sanya, China; 2 Ministry of Education Key Laboratory of Marine Genetics and Breeding, College of Marine Life Sciences, Ocean University of China, Qingdao, China

**Keywords:** Neuropeptide, MIP, Larval settlement, Gene pathway, Cilia-related genes, *Urechis unicinctus*

## Abstract

**Background:**

Larval settlement and metamorphosis represent critical events in the life history of marine benthic animals. Myoinhibitory peptide (MIP) plays a pivotal role in larval settlement of marine invertebrates. However, the molecular mechanisms of MIP involved in this process are not well understood.

**Results:**

In this study, we evaluated the effects of thirteen MIP mature peptides on triggering the larval settlement of *Urechis unicinctus* (Xenopneusta, Urechidae), and determined that MIP2 was the principal neuropeptide. Transcriptomic analysis was employed to identify differentially expressed genes (DEGs) between the MIP2-treated larvae and normal early-segmentation larvae. Both cAMP and calcium signaling pathways were enriched in the DEGs of the MIP2-treated larvae, and two neuropeptide receptor genes (*Spr*, *Fmrfar*) were up-regulated in the MIP2-treated larvae. The activation of the SPR-cAMP pathway by MIP2 was experimentally validated in HEK293T cells. Furthermore, fourteen cilia-related genes, including *Tctex1d2*, *Cfap45*, *Ift43*, *Ift74*, *Ift22*, *Cav1* and *Mns1*, etc. exhibited down-regulated expression in the MIP2-treated larvae. Whole-mount in situ hybridization identified two selected ciliary genes, *Tctex1d2* and *Cfap45*, were specially expressed in circumoral ciliary cells of the early-segmentation larvae. Knocking down *Tctex1d2* mRNA levels by in vivo RNA interference significantly increased the larval settlement rate.

**Conclusion:**

Our findings suggest that MIP2 inhibits the function of the cilia-related genes, such as *Tctex1d2*, through the SPR-cAMP-PKA pathway, thereby inducing larval settlement in *U. unicinctus*. The study contributes important data to the understanding of neuropeptide regulation in larval settlement.

**Supplementary Information:**

The online version contains supplementary material available at 10.1186/s12864-024-10228-y.

## Background

Most marine benthic invertebrates exhibit planktic larvae in their life cycle [1]. Along with these planktic larval development, they undergo settlement to sediment, rock or other substance surfaces, and then complete larval attachment and metamorphosis [2–4]. The transition of the larvae from a planktic to a benthic mode is universally recognized as a critical phase in their life cycle and plays a fundamental role in maintaining population quantity and community dynamics [5]. For commercial important aquatic animals, this phase holds particular significance in breeding by enhancing the rates of larval settlement and metamorphosis.

Larval settlement is a multifaceted process regulated by the interplay of biotic and abiotic factors [6–10]. Neuropeptides, as signaling molecules, have been reported to play a pivotal role in larval settlement and metamorphosis [11–15]. Myoinhibitory peptide (MIP)/allatostatin-B, belonging to the ancient W-amide neuropeptide superfamily, has been demonstrated to be involved in the larval settlement process of certain marine benthic invertebrates. In the annelid *Platynereis dumerilii*, exogenous application of neuropeptide MIP can prolong the closure time of cilia and induce larval settlement [16]. In the barnacle *Balanus amphitrite*, the mRNA expression pattern of *Mip* aligns with the larval settlement behavior, suggesting that MIP involves in the settlement process [17]. Additionally, MIP plays a vital role in regulating muscle contraction, hormone synthesis, metamorphosis, and ecdysis in arthropods [18–25]. SPR (Sex peptide receptor), originally identified as a sex peptide receptor regulating female reproductive behavior in *Drosophila melanogaster* [26, 27], is part of the MIP-SPR pathway, which has been identified to act on sleep homeostasis in *D. melanogaster* [28]. In the nematode *Caenorhabditis elegans*, MIP signaling through SPRR-2 (sex-peptide-receptor-related-2) modulates salt avoidance learning parallel to the insulin pathway [29]. Conzelmann et al. [16] demonstrated that MIP is expressed in chemosensory-neurosecretory cells of the apical organ in *P. dumerilii* and regulates larval settlement through signal transduction by SPR. Schmidt et al. [30] found that different MIP mature peptides derived from MIP precursors exhibit varying affinities with the receptor, resulting in different signal transmission activities. However, it remains unclear which downstream genes are regulated by the MIP-receptors pathway in inducing larval settlement.

The echiuran worm *Urechis unicinctus*, a commercially and ecologically important marine benthic invertebrate inhabiting in the intertidal zone, undergoes typical larval settlement and metamorphosis during its life cycle [31]. Due to its high nutritional and medicinal values, *U*. *unicinctus* is a prized resource in Asia [32, 33]. Hou et al. [34], through transcriptome analysis, revealed the involvement of the *Mip* precursor gene in the larval settlement and metamorphosis in *U*. *unicinctus*. Lu et al. [35] demonstrated that MIP1 can significantly trigger the settlement of *U. unicinctus* early-segmentation larvae. Bai et al. [36] identified two MIP receptors, MGIC (myoinhibitory peptide-gated ion channel) and SPR in *U. unicinctus*, and verified that *U. unicinctus* MIP1 can activate the SPR-cAMP pathway in HEK293T cells. In this study, we identified the principal MIP inducing early-segmentation larval settlement in *U. unicinctus*, and subsequently screened key downstream genes and pathways based on transcriptome and RT-qPCR analysis. Finally, we revealed the spatio-temporal expression characteristics of the cilia-related genes and verified the role of the ciliary gene *Tctex1d2* in larval settlement. Our work provides valuable scientific insights into the molecular mechanism of neuropeptides regulating larval settlement and the potential application of MIP inducing larval settlement in *U. unicinctus*.

## Materials and methods

### Animals and sampling

Adult *U. unicinctus* were collected from the intertidal area in Qinhuangdao, China. The acquisition of sperm and oocytes, artificial insemination and larval rearing procedures followed the methodology outlined by Wei et al. [37]. Zygotes (30 min post-fertilization), embryos including 4–8 cell embryos (1 h post-fertilization, hpf), blastulae (7 hpf), gastrulae (13 hpf), and larvae including early-trochophores (24 hpf), mid-trochophores (6 days post-fertilization, dpf), late-trochophores (8 dpf), early-segmentation larvae (12 dpf), late-segmentation larvae (21 dpf) and worm-shaped larvae (24 dpf) were collected, respectively. All samples used for whole-mount in situ hybridization were processed following the protocol outlined by Wei et al. [37].

### Assay of larval settlement treated by MIP mature peptides or PKA inhibitor

The MIP precursor protein of *U. unicinctus* consists of 475 amino acids with an approximate molecular weight of 60 KDa [34]. Thirteen MIP mature peptide sequences (Additional file 1) were predicted from a single gene (GenBank: MT162087.1), and the active MIPs with amidation in the carboxyl terminal of the last amino acid were synthesized by Sangon Biotechnology (Shanghai, China). In accordance with the methodology established by Lu et al. [35], the early-segmentation larvae (12 dpf) swimming in the upper water layer were collected and transferred to the forty-two glass tubes (diameter = 1.5 cm) with 11.5 cm height of solution, and the spatial distribution of larvae in the water layer was videoed continuously for 5 min. Each tube contained thirty larvae, and the solution in the tubes consisted of filtered seawater (FSW) with 10 µM MIP mature peptides in the experimental groups or the FSW without MIP in the control group. Three replicates were set for each group. The height (H) of each larva in the tube was measured, and the relative height (RH) of larvae was calculated using the formula RH = (H/11.5)%. Larval distribution in each group was quantified, and the MIP with maximum effect on inducing larval settlement was identified as the principal MIP mature peptide. Subsequently, the optimal concentration of the principal MIP was determined using the above experiment system with three concentrations (5, 10, 15 µM) of the principal MIP, respectively.

The PKA (protein kinase A) inhibitor experiment was carried out in six glass tubes (diameter = 2.5 cm) with a height of 10 cm of FSW. Each tube contained 200 early-segmentation larvae. The larvae in the experiment group were treated with FSW containing 0.5 µM H89, a PKA inhibitor (MedChemExpress, Princeton, USA), while the control group larvae were treated with FSW without the inhibitor. Three replicates were set for each group. After H89 treatment for 12 h, the proportion of larvae settling to the bottom of the glass tube was calculated. Meantime, the larvae were sampled and immediately frozen in liquid nitrogen before stored at − 80℃.

### Sampling and total RNA extraction of MIP2-treated larvae

The early-segmentation larvae were utilized for the experiment conducted in glass tubes (diameter = 2.5 cm) with a solution height of 11.5 cm. The solution of the treatment group was FSW with the optimal concentration (10 µM) of the principal MIP (MIP2), while that of the control group was FSW without MIP2. Each tube contained 200 larvae, and there were 3 replicates for each group. The larvae were collected at 1 min, 3 and 5 min of the experiment using 300-mesh sieves, respectively. After the solution containing MIP2 had been filtered, larvae on the sieve were quickly washed with FSW and transferred to the 1.5 mL RNase-free centrifuge tube using a pasteurized straw. Thereafter, the larvae were quickly centrifuged with a small centrifuge for 2–3 s, the supernatant was removed with a pasteurized straw, and then quickly frozen in liquid nitrogen. The sampling and frozen process can be completed within 10–15 s. Finally, the samples were stored at − 80℃.

Total RNAs were extracted from the stored larvae using Sparkzol Reagent (Sparkjade, Jinan, China) according to the manufacturer’s instructions. Total RNAs were assessed for amount and integrity using a NanoDrop spectrophotometer (Thermo Fisher Scientific, Waltham, USA) and 1% agar gel electrophoresis, and then stored at − 80℃.

### Transcriptome analysis

Total RNAs from the larvae treated for 3 min in 10 µM MIP2 and the control groups were used for transcriptome analysis. The mRNA was isolated from the total RNA (1 µg per sample) using oligo-dT beads (Qiagen, Hilden, Germany), and sequencing libraries were constructed using NEBNext® UltraTM RNA Library Prep Kit for Illumina® (NEB, Ipswich, USA) following the manufacturer’s protocols. The libraries (a total of 6 libraries from the two groups with 3 sample replicates) were respectively sequenced using Illumina NovaSeq 6000 (Illumina, San Diego, USA) by Novogene Company (Beijing, China) after their quality was assessed by Agilent 2100 bioanalyzer.

High-quality sequences (clean reads) were obtained from raw reads after eliminating the reads containing ambiguous bases, adapter sequences, etc. Trinity^v2.6.6^ was employed to jointly assemble all clean reads from the six libraries into unigenes [38]. BUSCO was used to assess the completeness of the transcriptome assembly [39]. Unigenes were annotated using NT, NR, KO, Pfam, SwissProt, GO, and KOG databases. GO annotation was conducted using Blast2GO^v2.5^, and functional classification was performed by WEGO [40].

The clean reads were mapped to the assembled whole transcriptome using software RSEM^v1.2.15^ [41]. The expression level of unigenes was quantified with TPM (Transcripts per million) values [42]. Differentially expressed genes (DEGs) were analyzed using DESeq2 with the threshold FDR (False Discovery Rate) < 0.05 and|log_2_Fold Change| > 1 [43]. GO function enrichment of DEGs was analyzed with GOseq^v1.10.0^ software. KEGG pathway enrichment analysis of the DEGs was conducted using KOBAS [44]. The Benjamin and Hochberg methods were employed to adjust the enrichment *p*-value, and significance was set with the adjusted *p* < 0.05.

### Plasmid construction and CRE luciferase activity assay

To elucidate the signal transduction mechanism of MIP2, *U. unicinctus* SPR was expressed in the HEK293T cells by co-transfection of a cyclic adenosine monophosphate (cAMP) response element-luciferase (CRE-Luc) reporter designed to target the cAMP pathway. The open reading frame (ORF) of *Spr* was isolated by PCR (Additional file 2) and subcloned into the expression vector pcDNA-3.1 (+) to generate the SPR expression vector. Using the expression vector, the HEK293T cells with stable expression of SPR was established.

The HEK293T cells were seeded in 24-well plates at a density of approximately 1 × 10^5^ cells/mL per well, followed by an overnight incubation for recuperation. Transfection was performed using Lipofectamine 2000 in Opti-MEM with pcDNA 3.1(+)-*Spr*, CRE-Luc, and a Renilla luciferase-expression vector, pRL-TK. The total amount of plasmids used in each co-transfection was 300 ng. Subsequently, the cells underwent a 36-h incubation at 37℃ in Dulbecco’s modified Eagle medium (DMEN) supplemented with 10% fetal bovine serum (FBS). The cells were maintained in FBS-free DMEM for a 16 h starvation followed by Forskolin (10 µM) (Yeasen, Shanghai, China) and MIP2 (10-10000 nM) treatment for an additional 6 h. Each treatment was set three replicates. Forskolin is an adenylate cyclase activator that directly interacts with the catalytic subunit of the enzyme to increase intracellular cAMP levels [45].

After a quick rinse with ice-cold phosphate-buffered saline (PBS), the cells were dissolved in a passive lysis buffer (Promega, Madison, USA). The resultant cell lysate was used to measure firefly luciferase and Renilla luciferase activities via a Dual-Glo luciferase assay kit (Yeasen, Shanghai, China). Transfection experiments were conducted in quadruplicate with cells cultured in individual wells.

### Real-time quantitative PCR (RT-qPCR)

Total RNAs were isolated from the stored samples following the procedure mentioned above. Subsequent to the removal of contaminant genomic DNA using 5 × gDNA digester Mix, the first strand cDNA was reversely transcribed using Hifair® III 1st Strand cDNA Synthesis SuperMix for qPCR (gDNA digester plus) from Yeasen, Shanghai, China. The cDNA sequences of target genes, including *Tctex1d2*, *Cfap45*, *Ift43*, *Ift74*, *Ift22*, *Cav1* and *Mns1* were obtained from *U. unicinctus* larval transcriptome. Fragments of these genes were respectively amplified with specific primers (Additional file 3), and a 123-bp fragment of *U. unicinctus Atpase* was served as an internal control gene [46]. RT-qPCR was conducted utilizing Hieff UNICON® Universal Blue SYBR Green Master Mix (Yeasen, Shanghai, China) on a qTOWER^3^G Real-Time PCR Thermal Cycler (Analytikjena, Jena, Germany). All reactions were carried out with three sample replicates and three technical replicates. Data were analyzed using the qPCRsoft^v4.1^ (Analytikjena, Jena, Germany), and the relative expression level of target mRNA was calculated using the 2^−ΔΔCt^ method.

### Whole-mount *in situ* hybridization (WISH)

Specific primers (Additional file 4) containing Sp6 or T7 promoter sequences were designed according to the cDNA sequences of *U. unicinctus Tctex1d2* and *Cfap45*. Digoxin labeled RNA probes of Sp6-sense and T7-antisense were synthesized using the DIG RNA Labeling Kit Sp6/T7 (Roche, Basel, Switzerland). WISH was conducted following the methodology outlined by Wei et al. [37]. Briefly, the samples were digested at 37℃ with 100 ng/mL proteinase K for 15 min (embryos), 20 min (trochophores) or 200 ng/mL proteinase K for 20 min (segmentation larvae), 15 min (worm-shaped larvae). Hybridization was carried out for 16 h at 60℃ using a probe concentration of 1 ng/µL in the hybridization buffer. The samples were incubated with the Anti-Digoxigenin-AP conjugate (Roche, Basel, Switzerland) at a 1: 2500 dilution for 16 h at 4℃, and stained in NBT/BCIP staining solution (Roche, Basel, Switzerland) in the dark for 0.5–1.5 h at room temperature. The results were observed and photographed using a Leica DM2500 LED microscope (Leica, Weztlar, Germany). Drawings and final panels were designed using Adobe Photoshop CS6 (San Jose, CA, USA).

### RNA interference (RNAi)

The cDNA fragments of *Tctex1d2* and *EGFP* (enhanced green fluorescent protein gene) were generated using the primers tagged with T7 sequence for dsRNA synthesis (Additional file 5). The quality of the PCR products was confirmed by 1% agar gel electrophoresis, followed by purification with the MolPure® PCR Purification Kit (Yeasen, Shanghai, China). The *Tctex1d2*-dsRNA and *EGFP*-dsRNA (as negative control) were synthesized using the MEGAscript® RNAi kit (Invitrogen, Waltham, USA) following the manufacturer’s protocols. The dsRNAs were assessed for quality and quantity using a NanoDrop spectrophotometer (Thermo Fisher Scientific, Waltham, USA) and 1% agar gel electrophoresis, and then stored at − 30℃ until use.

The larvae were reared following the method described by Wei et al. [37]. The early-segmentation larvae (10 dpf) were randomly divided into three groups: the *Tctex1d2*-dsRNA group, *EGFP*-dsRNA group, and blank control group. Each group had three replicates, with 170 larvae in each replicate. The larvae of the *Tctex1d2*-dsRNA group and *EGFP*-dsRNA group were initially soaked in 3 mL FSW with a final dsRNA concentration of 50 nmol/L for 12 h, and then respectively transferred to glass tubes containing 50 mL FSW without dsRNA for 60 h. For the blank control group, the larvae were treated in 3 mL FSW without any dsRNA, and other conditions were the same as the dsRNA treatment groups. At the end of larval treatment, number of the settled larvae was recorded. The settled larvae were defined as those attaching to the bottom surface of the glass tubes and unable to swim into the seawater layer. The settlement rate was calculated using the formula: the number of settled larvae / the total number of larvae * 100%.

To assess the efficiency of gene knockdown, the larvae were collected at 72 h post dsRNA treatment and stored at − 80℃ after immediately frozen in liquid nitrogen. Total RNAs from the nine samples across the three groups were respectively extracted using Trizol reagent (Yeasen, Shanghai, China). The cDNA synthesis was performed and the expression level of *Tctex1d2* in the larvae was determined by RT-qPCR to assess the RNAi effect.

### Statistical analysis

All data were presented as mean ± SEM. One-way analysis of variance (ANOVA) followed by Duncan’s multiple-range test and independent samples T-test, using SPSS^v22^ software (SPSS Inc., Chicago, USA) was employed to determine significant differences between means. Statistical significance was set at *p* < 0.05.

## Results

### MIP2 as the principal MIP mature peptide in triggering larval settlement of ***U. unicinctus***

**Fig. 1:**
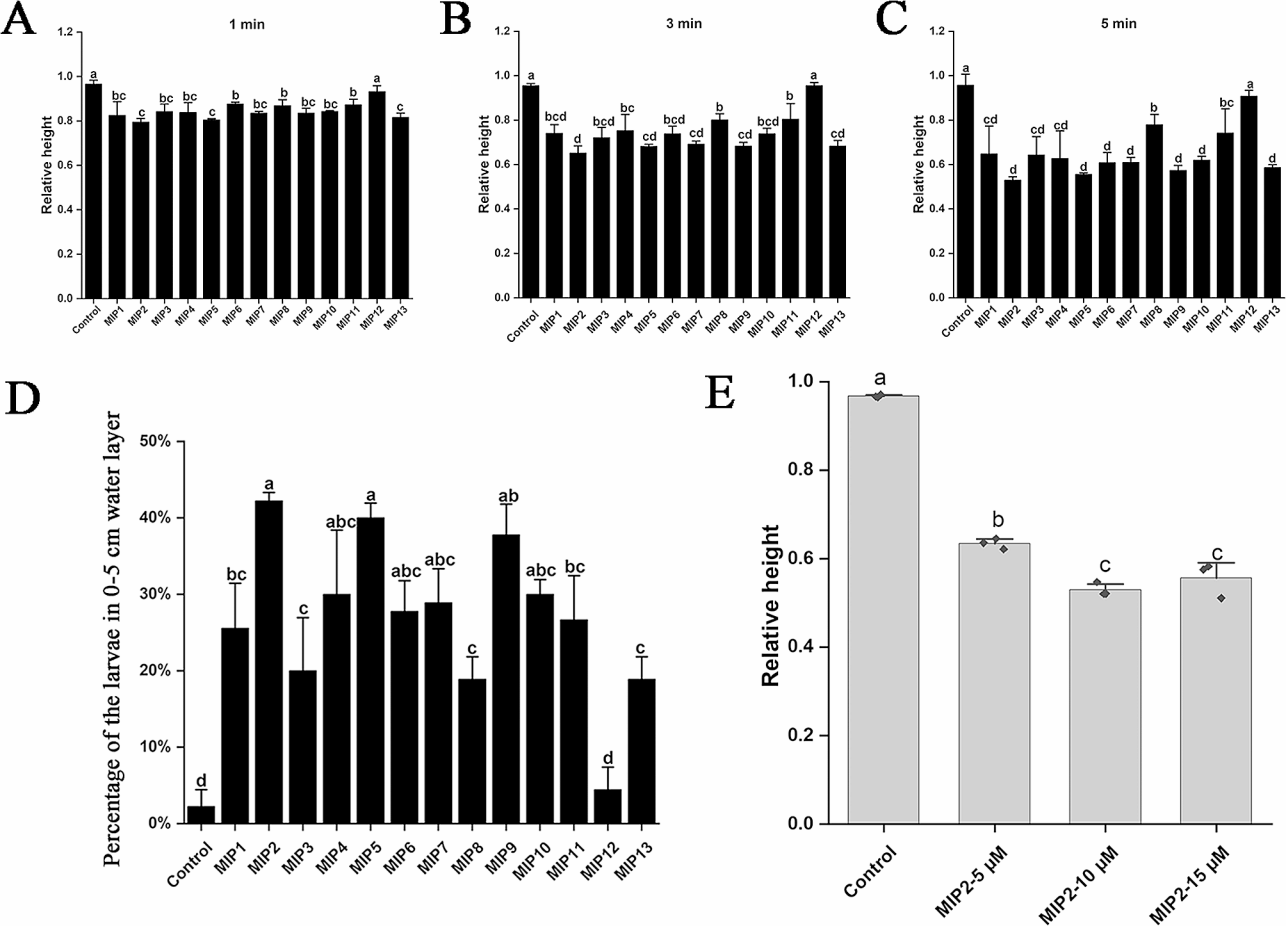
MIP-induced larval settlement assays. **A-C**: Relative height of the larvae treated with 13 MIPs at 1 min, 3 and 5 min; **D**: Percentage of the larvae in 0–5 cm water layer after treated by 13 MIPs for 5 min, respectively; **E**: Relative height of the larvae treated by MIP2 at the different concentrations for 5 min. Each rhombus represents a data. All data are represented as the mean ± SEM from three biological replicates. Different letters indicate significant differences (*p* < 0.05)

We synthesized 13 MIP mature peptides and detected the effect of these MIPs on triggering the early-segmentation larval settlement (Additional file 6 and 7). The results showed that 12 MIPs (MIP1-11 and MIP13) significantly induce the larval settlement, with the exception for MIP12 (Fig. 1A-C). In MIP2 treatment group, the average relative height of larvae in the water layer was always the lowest, exhibiting reduction of 18.80% (1 min), 32.34% (3 min) and 46.18% (5 min) in comparison to that of the control group, respectively. Following closely was MIP5, which displayed reductions of 18.42% (1 min), 29.24% (3 min) and 43.55% (5 min) relative to the control group, respectively. Moreover, the percentage of larvae in the lower water layer (0–5 cm) was the largest in MIP2 group at the 5 min treatment, constituting 42.22% (Fig. 1D and Additional file 8). Based on the relative height of the larvae in the water layer and the amount of the larvae in the lower water layer, MIP2 emerged as the principal MIP mature peptide in triggering larval settlement of *U. unicinctus*. Up subjecting the early-segmentation larvae to varying concentrations of MIP2, the larval relative height (RH) in the 10 or 15 µM MIP2 groups was significantly lower than that of 5 µM MIP2 group and control group. Notably, no significant difference was observed between the 10 µM MIP2 and 15 µM MIP2 groups (Fig. 1E). Consequently, the optimal concentration for MIP2-induced early-segmentation larval settlement was determined to be 10 µM based on the lowest RH value. Intriguingly, we observed that after 7 h of MIP2 treatment, the circumoral cilia of the larvae underwent closure, in stark contrast to the dispersed arrangement observed in the control larvae (Additional file 9).

### Identification and enrichment analysis of DEGs in transcriptomes between MIP2-treated and control larvae of ***U. unicinctus***

Six cDNA libraries were constructed from both the control and MIP2-treated larvae, yielding a total of 132.82 Mb raw reads, and subsequent processing resulted in 131.76 Mb clean reads. The assembly produced 43,034 unigenes with an average length of 1476 bp and N50 of 2581 bp (Tables 1 and 2). The RNA-seq data have been deposited in the NCBI database with the accession number of PRJNA1027755.

**Table 1:** Overview of sequencing reads from six larval transcriptomes of *U. unicinctus*

Sample	Raw reads	Clean reads	Clean bases	Error (%)	Q20 (%)	Q30 (%)	GC (%)
Control_1	24,087,883	23,849,793	7.15 G	0.02	98.04	94.18	43.35
Control_2	22,314,173	22,188,495	6.66 G	0.03	97.59	93.07	42.03
Control_3	22,210,116	22,083,247	6.62 G	0.03	97.68	93.33	42.32
MIP2_1	22,335,435	22,160,119	6.65 G	0.02	98.41	94.99	45.66
MIP2_2	21,071,633	20,896,341	6.27 G	0.03	97.92	93.95	43.5
MIP3_3	20,804,832	20,583,068	6.17 G	0.02	98.48	95.14	45.17

Q20/Q30: percentage of the bases with a quality value larger than 20 or 30.

**Table 2:** Length distribution of the assembled transcripts and unigenes in *U. unicinctus* larval transcriptomes

Type	Min Length	Mean Length	Median Length	Max Length	N50	N90	Total Nucleotides
Transcripts	301	1806	1185	52,776	2910	790	186,742,931
Unigenes	301	1476	781	52,776	2581	542	63,505,589

N50/N90: the shortest sequence length at 50%/90% of the total length of the spliced transcripts.

**Fig. 2:**
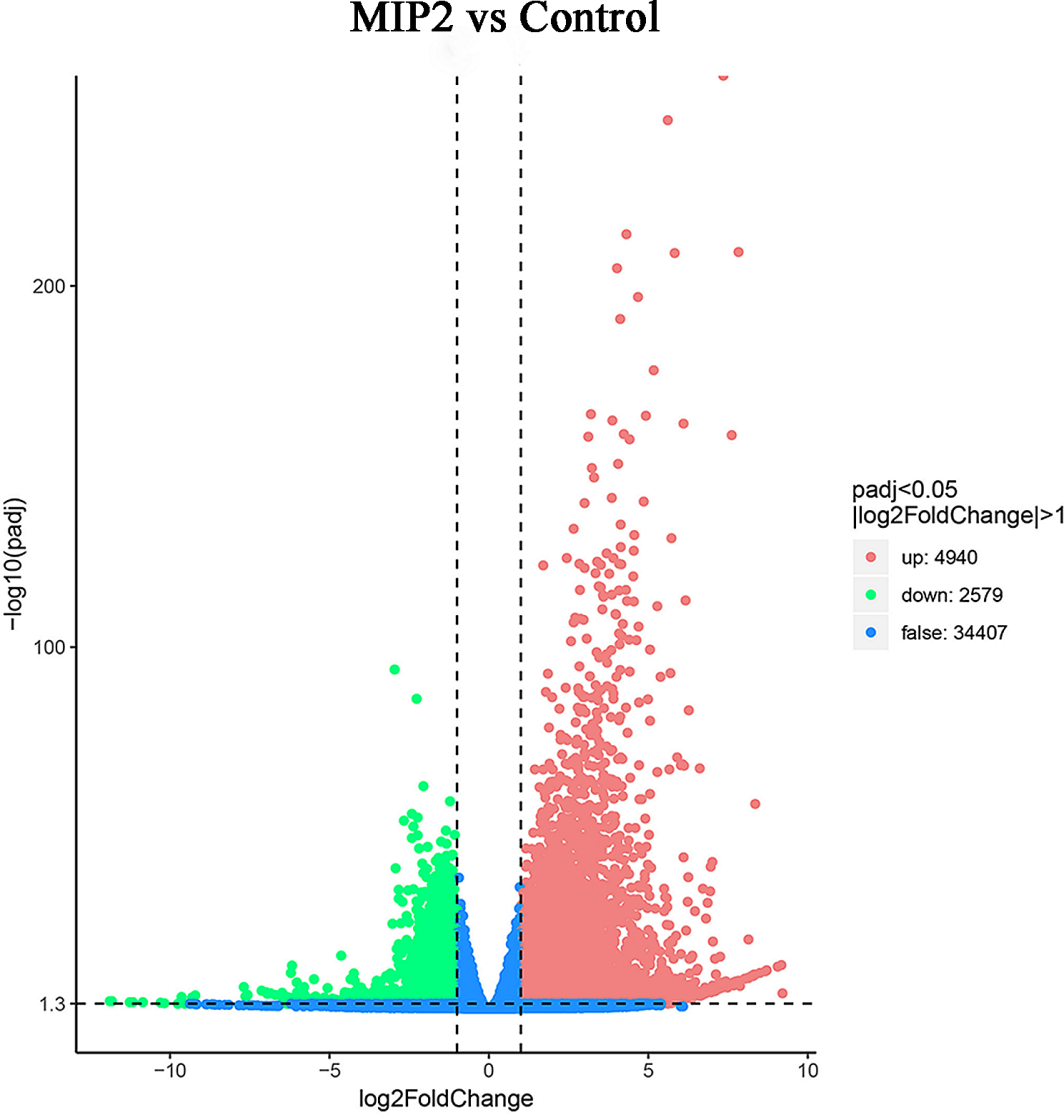
Volcanoplot illustrating the differentially expression of unigenes between the control group larvae and those treated with MIP2 for 3 min. Pink and green spots indicate the differentially expressed unigenes, while blue spots indicate no differentially expression

**Fig. 3:**
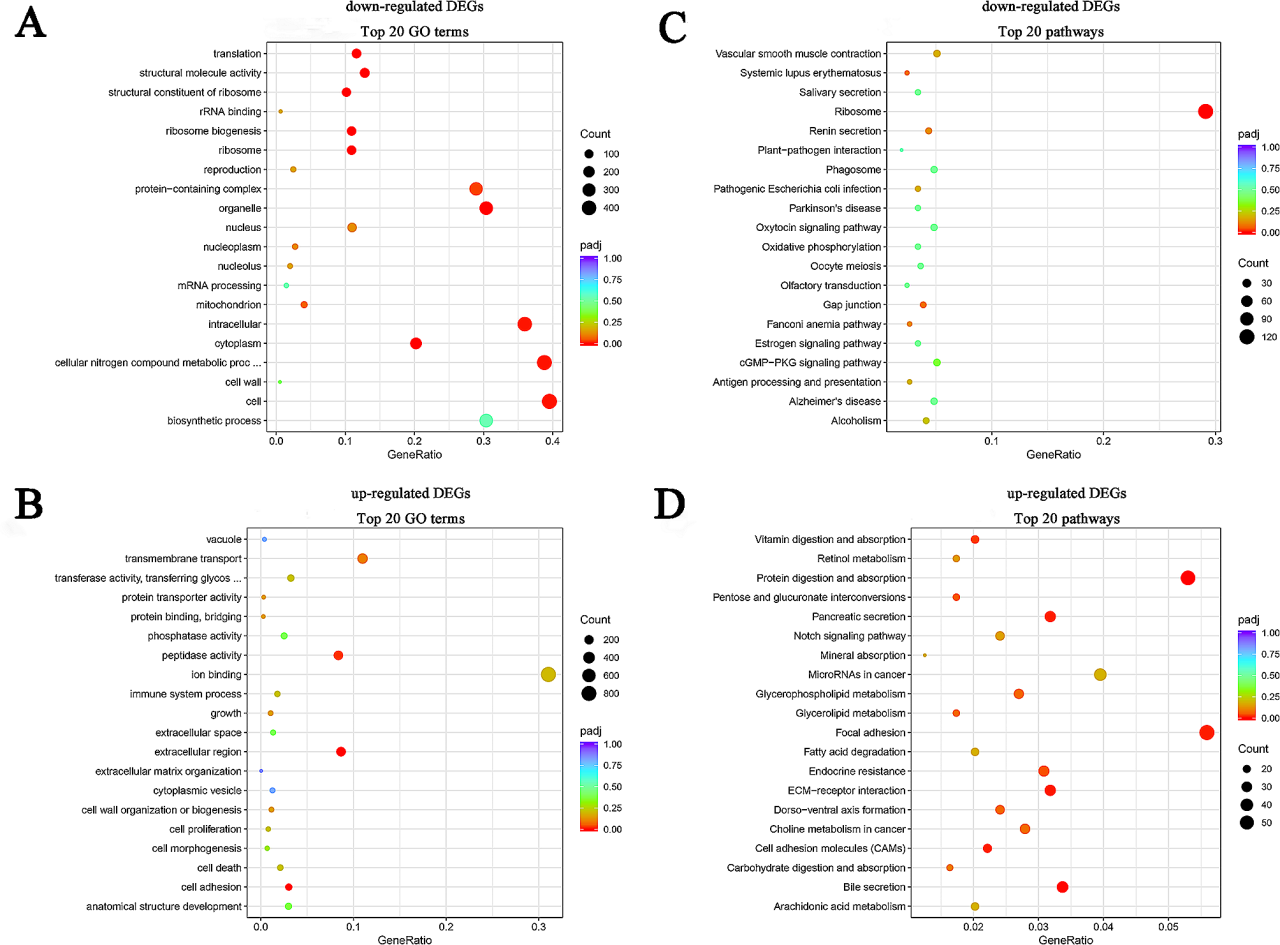
Gene Ontology (GO) and Kyoto Encyclopedia of Genes and Genomes (KEGG) enrichment analysis of the differentially expressed genes (DEGs). **A**: GO enrichment analysis of the down-regulated DEGs; **B**: GO enrichment analysis of the up-regulated DEGs; **C**: KEGG enrichment analysis of the down-regulated DEGs; **D**: KEGG enrichment analysis of the up-regulated DEGs

A comprehensive analysis revealed 7519 DEGs, comprising 4940 up-regulated and 2579 down-regulated genes in MIP2-treated larvae (Fig. 2). Notably, several genes associated with neuropeptide receptor or key signaling molecules (Additional file 10) and fourteen cilia-related genes (Additional file 11) were contained in the down-regulated DEGs. GO enrichment analysis of the down-regulated DEGs identified eleven significantly enriched terms, such as structural constituent of ribosome, organelle, and protein-containing complex (Fig. 3A). Conversely, only three GO terms, namely cell adhesion, extracellular region and peptidase activity were significantly enriched in the up-regulated DEGs (Fig. 3B).

KEGG enrichment analysis showed that the down-regulated and the up-regulated genes were enriched in 241 and 327 signaling pathways, respectively (Additional file 12 and 13). Among the down-regulated DEGs, ribosome was the sole significantly enriched signaling pathway (Fig. 3C), while the up-regulated DEGs featured nine enriched pathways, including protein digestion and absorption (Fig. 3D). Interestingly, several pathways were concurrently enriched in both down-regulated DEGs and up-regulated DEGs, such as cAMP, MAPK, AMPK, Notch, Rap1 and calcium signaling pathways (Additional file 12 and 13).

### MIP2 activates SPR in a dose-dependent manner

**Fig. 4:**
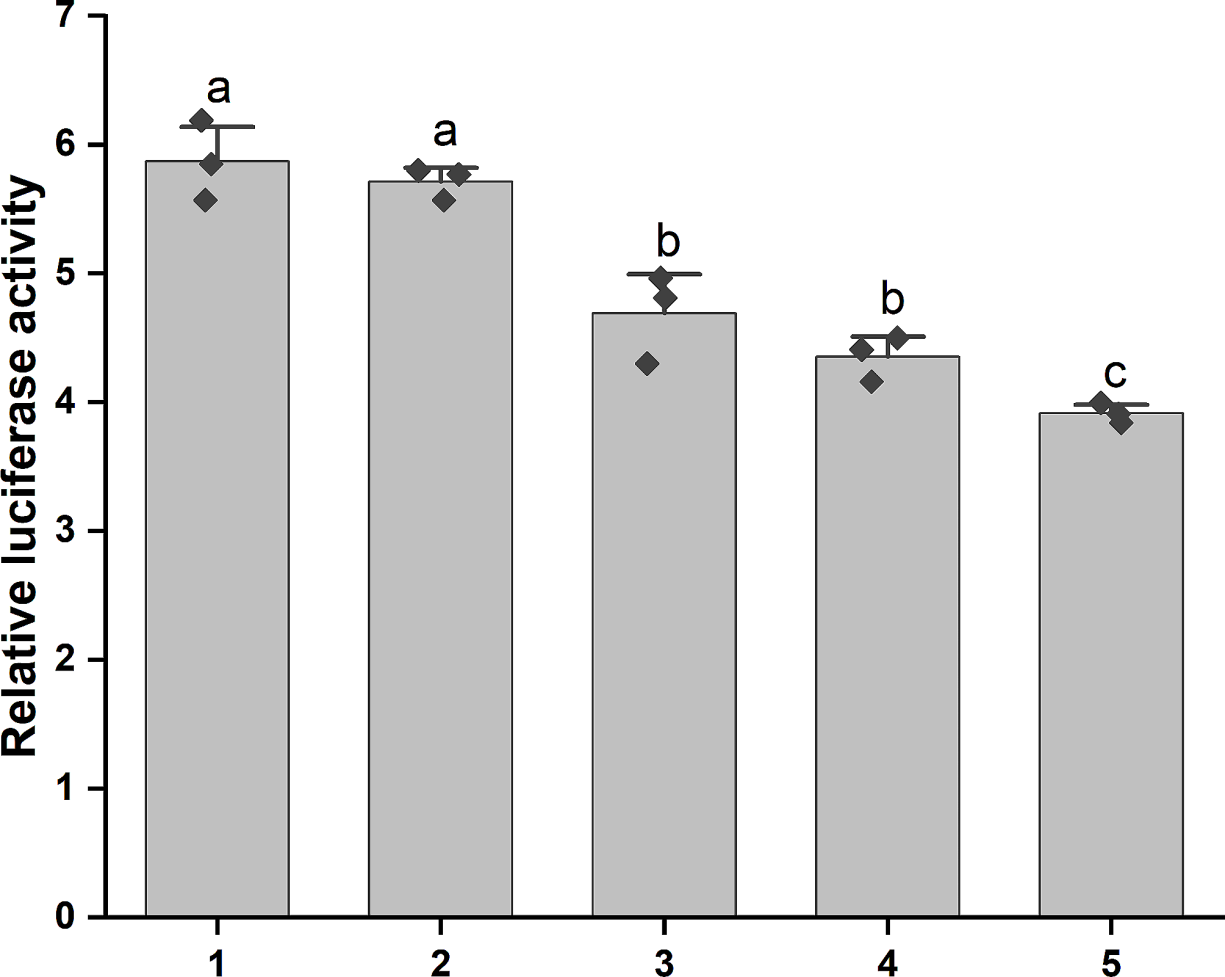
CRE-driven luciferase activity in HEK293T cells treated by MIP2. 1: the Forskolin treatment group with 10 µM; 2–5: MIP2 treatment group with 10, 100, 1000, 10,000 nM + 10 µM Forskolin, respectively. Each rhombus represents a data. Different letters indicate significant differences (*p* < 0.05)

We found that the expression of the neuropeptide receptor gene *Spr* was significantly increased in MIP2-treated larvae, and previous studies have demonstrated that *U. unicinctus* MIP1 can activate SPR [36]. To examine the effects of MIP2 on the receptor SPR, HEK293T cells were employed, wherein *Spr*-ORF was co-transfected with the CRE-Luc reporter system to evaluate the potential MIP2 signaling pathway coupled to SPR receptor in *U. unicinctus*. The result showed that *U. unicinctus* MIP2 exhibited inhibitory effects on Forskolin-stimulated CRE-Luc activity in a dose-dependent manner in HEK293T cells (Fig. 4).

### Expression characteristics of ciliary genes and settlement rate of the early-segmentation larvae treated by MIP2 and H89

**Fig. 5:**
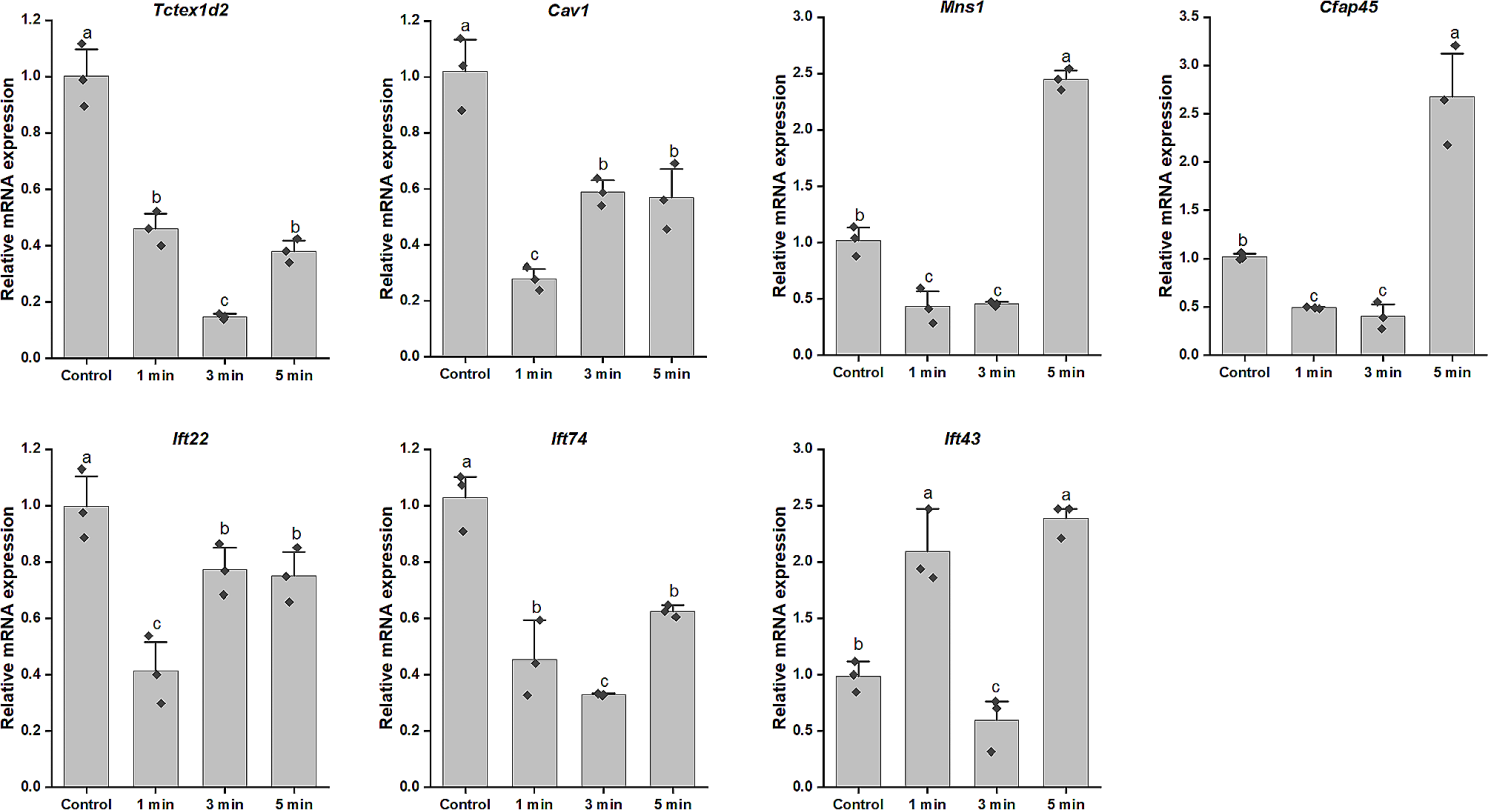
Relative mRNA level of seven cilia-related genes in the MIP2-treated larvae at different times. Each rhombus represents a data. Different letters indicate significant differences (*p* < 0.05)

Seven cilia-related genes were selected from the above fourteen genes, and their expression dynamics in early-segmentation larvae treated with MIP2 were scrutinized using RT-qPCR. Three expression patterns emerged among these genes. Specifically, the mRNA levels of the four genes, *Tctex1d2*, *Ift74*, *Cav1* and *Ift22* were consistently down-regulated significantly at 1 min, 3 and 5 min post MIP2 treatment compared to that of the control group. In contrast, *Cfap45* and *Mns1* demonstrated significantly down-regulated at 1 and 3 min but a pronounced up-regulation at 5 min. Intriguingly, *Ift43* exhibited significant down-regulation only at 3 min, but noteworthy up-regulation at 1 and 5 min (Fig. 5). The results of RT-qPCR were consistent with that of the transcriptome, suggesting that responses of these genes to MIP2 are distinctly different in both intensity and timing.

**Fig. 6:**
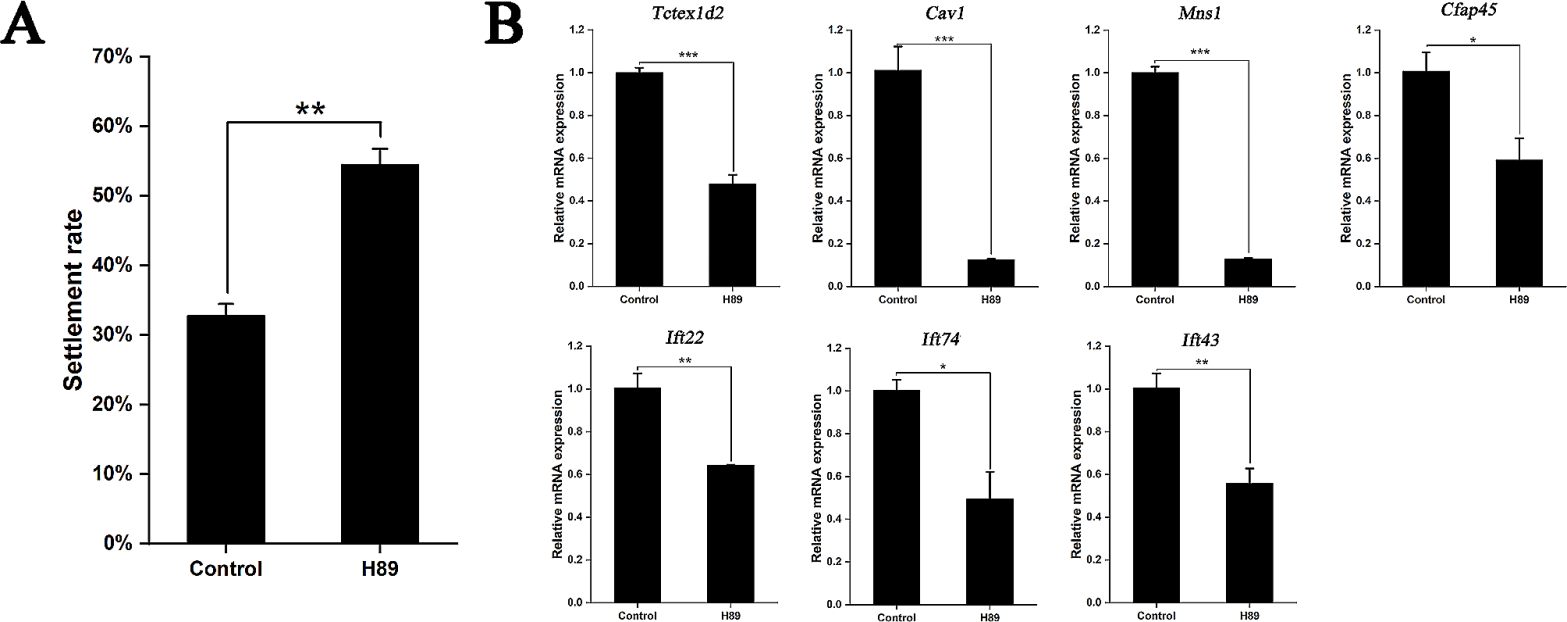
Settlement rate and target gene mRNA relative levels of the larvae treated with H89. **A**: Settlement rate of the larvae treated with H89. **B**: Relative mRNA level of seven ciliary genes in the larvae treated with H89. Data are represented as the mean ± SEM from three biological replicates. Asterisks indicate significant differences between H89 groups and the control group (* *p* < 0.05; ** *p* < 0.01; *** *p* < 0.001)

H89 is the cAMP-dependent PKA inhibitor, which regulates the expression of downstream genes by inhibiting the PKA activity to reduce the phosphorylation of target proteins. After H89 treated, the larval settlement rate increased significantly by 21.7% compared to the control group (Fig. 6A). The results of RT-qPCR showed that all the above seven cilia-related genes were significantly down-regulated in the early-segmentation larvae after H89 treatment (Fig. 6B).

### Temporal and spatial expressions of ***Tctex1d2*** and ***Cfap45*** in ***U. unicinctus*** embryos and larvae

Two representative genes, *Tctex1d2* and *Cfap45*, exhibiting disparate response patterns to MIP2, were selected for a detailed exploration of their expression patterns in *U. unicinctus* embryos and larvae. FPKM values retrieved from transcriptome data (NCBI accession number: PRJNA485379) revealed nuanced patterns. *Tctex1d2* exhibited a gradual increase from 2 to 8 cell embryo to trochophore, followed by a decline from late-trochophore to worm-shaped larva (Additional file 14). In contrast, *Cfap45* demonstrated its highest expression in 2–8 cell embryo, diminishing significantly in multicellular embryo, blastula and gastrula. Notably, *Cfap45* expression spiked in trochophore, and gradually decreased from late-trochophore to worm-shaped larva (Additional file 14).

**Fig. 7:**
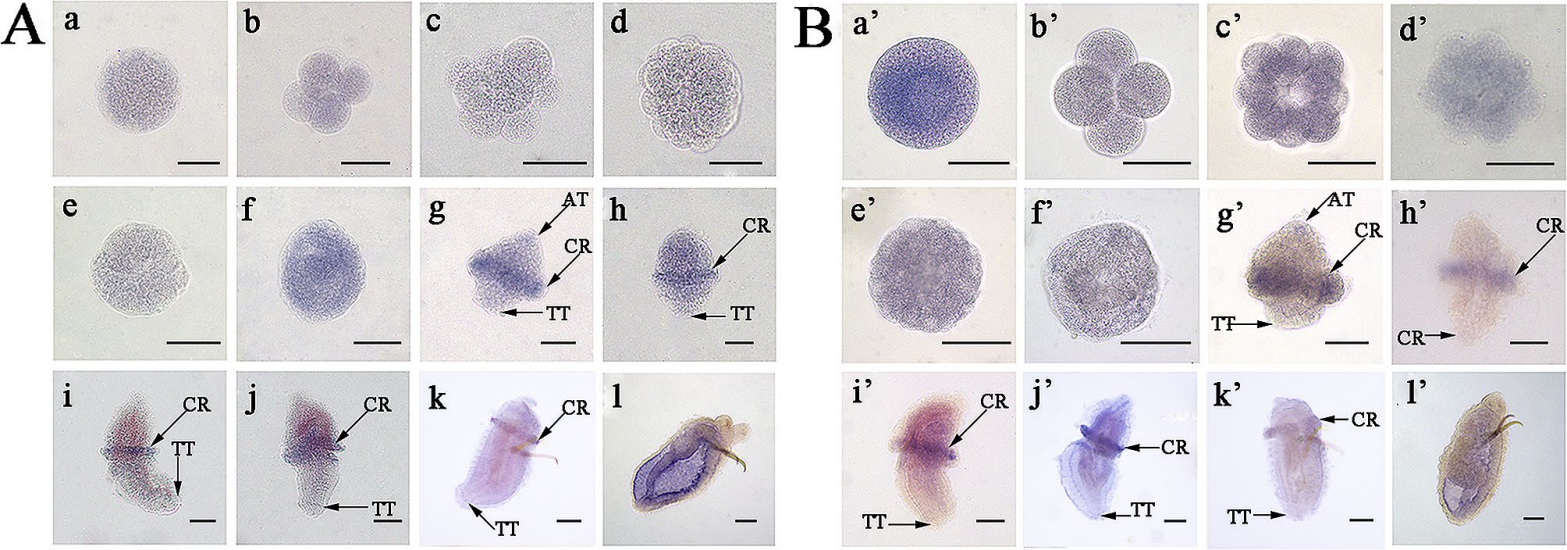
Location of *Tctex1d2* and *Cfap45* mRNA in *U*. *unicinctus* embryos and larvae detected by Whole-mount in situ hybridization. **A**: *Tctex1d2*; **B**: *Cfap45*; a and a’: zygote; b and b’: 4-cell embryo; c and c’: 8-cell embryo; d and d’: multicellular embryo; e and e’: blastula; f and f’: gastrula; g and g’: early-trochophore; h and h’: mid-trochophore; i and i’: late-trochophore; j and j’: early-segmentation larva; k and k’: late-segmentation larva; l and l’: worm-shaped larva. Blue indicates the positive signal. AT: apical ciliary tuft; CR: circumoral ciliary ring; TT: telotroch. All scales are 50 μm

The WISH results further validated these expression patterns. Obvious positive signals of *Tctex1d2* mRNA initially appeared in the gastrula, displaying a dispersed distribution (Fig. 7A a-f). After hatching, the strong positive signals of *Tctex1d2* mRNA were mainly located in the circumoral ciliary cells of trochophores or segmentation larvae (Fig. 7A g-k). In the worm-shaped larva, the *Tctex1d2* positive signals were observed in the digestive tract and the inner side of the body wall (Fig. 7A l). Positive signals of *Cfap45* mRNA were diffuse in the embryos at the different stages, with the strongest signal observed in zygote (Fig. 7B a’-f’). In the trochophore and segmentation larvae, the positive signals were predominantly located in the circumoral ciliary cells and the apical tuft cells of early-trochophore larvae (Fig. 7B g’-k’). Similar to *Tctex1d2*, in the worm-shaped larva, *Cfap45* mRNA signals were also observed in the digestive tract and the inner body wall (Fig. 7B l’). No positive signal was detected in embryos and larvae treated with *Tctex1d2* or *Cfap45* negative probes (Additional file 15).

### ***Tctex1d2*** mRNA knockdown promotes settlement of the early-segmentation larvae

**Fig. 8:**
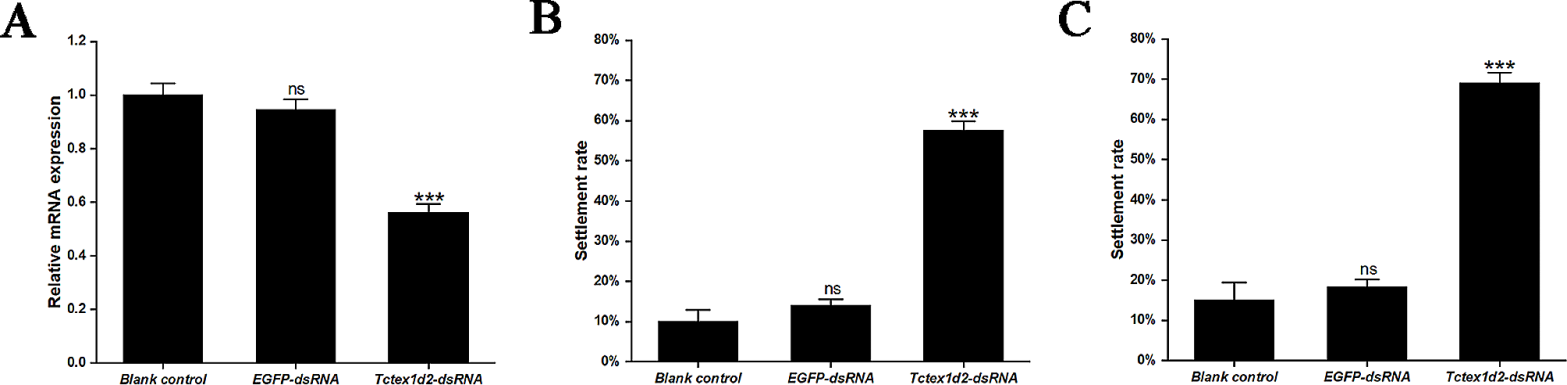
Gene knockdown efficiency and the settlement rate of the larvae after RNAi. **A**: Gene knockdown efficiency of *Tctex1d2* in dsRNA-treated larvae for 72 h. **B**: Larval settlement rate at 48 h of RNAi. **C**: Larval settlement rate at 72 h of RNAi. Data are represented as the mean ± SEM from three biological replicates. Asterisks indicate significant differences between *Tctex1d2*-dsRNA group and blank control group (*** *p* < 0.001). “ns” indicates no significant differences between *EGFP*-dsRNA groups and blank control group

RT-qPCR analyses demonstrated a significant reduction (*p* < 0.001) in the expression level of the ciliary gene *Tctex1d2* in the *Tctex1d2*-dsRNA group after RNAi for 72 h, representing a 43.76% decrease compared to the blank control group (Fig. 8A). Importantly, the settlement rate of larvae in the *Tctex1d2*-dsRNA group increased extremely significantly, reaching 57.65% (48 h after RNAi) and 69.19% (72 h after RNAi), respectively. In contrast, the settlement rates were only 10.06% (48 h) and 15.09% (72 h) in the blank control group, and 14.16% (48 h) and 18.40% (72 h) in the *EGFP*-dsRNA group (Fig. 8B-C). These findings underscore the significant impact of *Tctex1d2* knockdown in promoting the settlement of early-segmentation larvae.

## Discussion

### The effect of MIPs on larval settlement in ***U. unicinctus*** correlates with receptor affinity

The process of larval settlement is intricately regulated by diverse internal and external signals, exerting a profound influence on the normal development and survival of animals. Neuropeptides have been recognized as a critical role in this intricate process [12, 14, 47, 48]. Maturation of neuropeptides involves the cleaving of neuropeptide precursors, with various mature peptides from the same precursor exhibiting distinct functions [30, 49]. MIP has been revealed in promoting larval settlement in several marine invertebrates. For example, MIP1 and MIP4-MIP7 in *P*. *dumerilii* significantly induce the downward vertical movement of trochophore at the concentration of 5 µM [16]. In *Tenebrio molitor*, MIP5 stimulates contractile activity in beetle oviduct muscles at a concentration of 10 nM [50]. In *U*. *unicinctus*, we revealed that 12 MIP mature peptides, excluding MIP12, significantly induce the early-segmentation larval settlement. Among them, MIP2 emerged as the principal MIP, with the most effective dose being 10 µM (Fig. 1E). This suggests the similar characteristics, despite variations in the MIP concentration among the different species [16].

Schmidt et al. [30] observed variations in the affinity of 11 MIPs to their receptors, MAG (myoinhibitory peptide activated GPCR) and MGIC in *P*. *dumerilii*. MIP6, for example, activates MAG with EC_50_ values in the nanomolar range, being the most potent ligand. In contrast, MIP2 is a less effective ligand, activating MAG at micromolar concentration. Notably, for MIGC, MIP2 emerges as the most potent ligand. This divergence in the effects of different MIP mature peptides in *U. unicinctus* may stem from variations in receptor affinity. This, in turn, may lead to the activation of associated signaling pathways, resulting in the differences in the effects on downstream target genes. Consistently, in both *P*. *dumerilii* and *U*. *unicinctus*, MIP treatment triggered larval settlement and induced alterations in larval ciliary behavior (Additional file 9), underscoring the universality of MIP in inducing larval settlement of marine benthic invertebrates.

### Multiple signaling pathways involved in MIP2-induced larval settlement

In this study, a total of 7519 DEGs (17.47% of all unigenes) were identified in the transcriptomes of larvae subjected to the MIP2 treatment compared to the control group. Subsequently, a multitude of signaling pathways exhibited enrichment in both down-regulated and up-regulated DEGs (Additional file 12 and 13). Larval settlement caused by short-term neuropeptide treatment is prevalent in marine benthic invertebrates [16, 35, 47], and larval settlement is a complex process, mediated by diverse signaling pathways [51]. Furthermore, changes in phenotype and numerous gene expression caused by the treatment of exogenous stimuli within minutes have been reported in other species [52–54]. Although the mechanism of rapid biological response to this exogenous stimulus has not been fully elucidated.

Neuropeptides conventionally engage the G-proteincoupled receptor signaling pathway. Conzelmann et al. [16] proposed that MIP7 induces prolonged and frequent ciliary arrests in *P*. *dumerilii* trochophore, utilizing the conserved G-proteincoupled receptor signaling pathway to regulate the larval settlement. Additionally, Bai et al. [36] verified the capability of *U. unicinctus* MIP1 to activate its SPR receptor, thereby reducing the intracellular cAMP concentration in HEK293T cells. Our findings revealed that MIP2 induced a decline in intracellular cAMP concentration by activating the SPR receptor, as evidenced by the use of a CRE luciferase reporter plasmid. It is noteworthy that ADCY3 (adenylate cyclase 3) mediates ATP to cAMP conversion [55], and cAMP as a second messenger activates PKA pathway by stimulating PKA catalytic activity. Conversely, PDE4 (phosphodiesterase 4) hydrolyzes cAMP [56]. Consequently, the observed decrease in *Adcy3* expression or an increase in *Pde4* expression following MIP2 treatment signified a reduction in cAMP concentration (Additional file 16), thereby inhibiting the PKA activity. These findings strongly supported the assertion that MIP2 induces the larval settlement by the SPR-cAMP pathway.

When treating the early-segmentation larvae with the PKA inhibitor (H89), we observed a significant increase in larval settlement rate and a significant reduction in the expression levels of ciliary genes (Fig. 6). This aligned with the results following MIP2 treatment, suggesting that MIP2 regulates downstream genes by inhibiting PKA, thus delineating the SPR-cAMP-PKA pathway. It has been reported that peptides bind to GPCRs to activate phospholipase C (PLC), cause changes in Ca^2+^ concentration, activate Calmodulin-dependent protein kinase (CaMK), and thus regulate downstream effector genes [57, 58]. In this study, a significant elevation in mRNA level of the neuropeptide FMRFamide receptor gene (*Fmrfar*) and calcium signaling pathway related genes (*Calm*, *Camta1*, *Camkk1*, *CamkII* and *Plcb4*) was noted post MIP2 treatment. Given that the neuropeptide FMRFamide receptor has been associated with stimulating Ca^2+^ signals and modulating the activity of dopaminergic neurons in *D. melanogaster*, and that CaMKII serves as a downstream signaling component of FMRFamide receptor [59]. Therefore, we speculated that MIP2 holds the potential to activate the FMRFamide receptor. This activation triggers PLC in turn, expediting the decomposition of PIP2 (phosphatidylinositol(4,5)bisphosphate), resulting in the release of Ca^2+^ from the endoplasmic reticulum and an increase in the expression of the calmodulin gene *Calm*, then CaMKII is further activated to regulate the expression of downstream ciliary genes.

Conzelmann et al. [47] postulated the thrust exerted on the body is proportional to the beat frequency of cilia during larval swimming, where alternating phases of spontaneous beating and closure of cilia govern swimming depth in planktonic larvae. The coordinated beating of cilia in eukaryotes is regulated by axonemal heavy chain dynein ATPases, which are motor proteins generating force along microtubules through ATP hydrolysis [60]. Our results elucidate KEGG terms mainly involved in energy metabolism, encompassing protein digestion and absorption, glycerophospholipid metabolism, carbohydrate digestion and absorption, pentose and glucuronate interconversions, linoleic acid metabolism, arachidonic acid metabolism, glycolysis/gluconeogenesis, fat digestion and absorption, fatty acid degradation, fructose and mannose metabolism signaling pathways (Additional file 12 and 13). This metabolic pattern mirrors observations in larval settlement progress of other marine invertebrates, such as the barnacle *B*. *amphitrite* [61–64] and Fujian oyster *Crassostrea angulate* [65]. Moreover, we also noticed that enrichment in several signaling pathways (MAPK, AMPK, Notch, Rap1 and Foxo) associated with signal transduction (Additional file 12 and 13), known for their conservation in participating in larval settlement of marine invertebrates [66–69]. AMPK, a cellular energy sensor pivotal in maintaining the energy balance of the cell and the whole body [70], and FoxO, involved in crucial cellular processes like metabolism, apoptosis, proliferation and survival, may potentially play a role in MIP2-induced larval settlement of *U. unicinctus*. The adhesion process, crucial for survival and settlement in sessile organisms, was also implicated in our study. Focal-adhesion signaling pathway, including genes *RHO* and *RHO1*, and ECM-receptor interaction, were significantly enriched (*p* < 0.05), suggesting the involvement of adhesion alterations in *U. unicinctus* larval settlement.

In conclusion, the induction of larval settlement in *U. unicinctus* by MIP2 is a complex physiological phenomenon, involving many interconnected biological processes spanning organismal systems, metabolism and environmental information processing, among others.

### MIP promotes larval settlement by regulating the ciliary gene expression

Cilia, ubiquitous organelle presenting on the larval surface of various marine invertebrates, play a crucial role in facilitating larval swimming and aiding in feeding processes [71]. In *U*. *unicinctus*, cilia were initially observed as uniform distributed short structures on the body surface in the blastula under light microscope (Additional file 17: Fig. S7E-F). Subsequently, in the early-trochophore, cilia on the body surface manifested mainly as an apical ciliary tuft, circumoral ciliary ring and telotroch. In the mid-trochophore, the apical ciliary tuft disappears (Additional file 17: Fig. S7G-H). After that, in late-trochophore, early-segmentation larva and segmentation larva, cilia were mainly in the form of circumoral ciliary ring and telotroch (Additional file 17: Fig. S7I-K). Eventually, in the worm-shaped larva, all cilia on the body surface disappeared completely (Additional file 17: Fig. S7L) [72]. The settlement process will be initiated normally during the early-segmentation larva in *U*. *unicinctus* (Additional file 17: Fig. S7J), but this natural settlement was extremely slow and usually took 2–3 days from the water surface to the bottom. While, there was significant settlement in the MIP2-treated early-segmentation larvae (Fig. 1A-C). The activity of cilia is responsive to environmental cues and is generally regulated by nervous system [73–75]. Neuropeptides known to regulate larval settlement by influencing ciliary activity have been documented in several species, including *P. dumerilii* [46], *Crepidula fornicate* [76], *T*. *transversa* [77], and *Lineus longissimus* [78]. Although the diversity in effects (activation or inhibition) of different neuropeptides on ciliary beat frequency, it remains unclear which cilia-related genes are specifically regulated during the process.

In this study, utilizing the transcriptome data from the larvae in both the MIP2 treated and control groups, we identified fourteen previously reported cilia-related genes (Additional file 11). These genes mainly function in ciliogenesis/assembly and ciliary beating (Table 3). Furthermore, we identified the predominant localization of mRNA for two selected ciliary genes, *Tctex1d2* and *Cfap45*, in circumoral ciliary cells of *U. unicinctus* early-segmentation larvae by WISH (Fig. 7). Additionally, SPR, a MIP receptor which was expressed in the ciliary zone of larvae [36] was also identified to increased significantly in MIP2-treated larvae (Additional file 10), indicating that MIP2 may regulate ciliary gene expression through SPR. Knocking down the mRNA level of *Tctex1d2* by RNAi resulted in a significantly increased for the larval settlement rates, by 3.59 times at 48 h and 4.73 times at 72 h compared to the blank control group (Fig. 8). TCTEX1D2, recognized as a light chain of the dynein-2 complex, collaborates with the IFT-A complex to facilitate retrograde ciliary protein transport [96]. Perturbing the *Tctex1d2* gene, either through knockout or mutation, typically reads to phenotypes such as short cilia [97], delayed ciliogenesis [98], or a reduction in ciliated cells [99] in mammalian cells and zebrafish. CFAP45 (Cilia- and flagella-associated protein 45) supports ciliary and flagellar beating in mammalian through an adenine nucleotide homeostasis module. In *Cfap45*-deficient embryos and cells, the ciliary beat frequency [60] or the ciliary stability [79] is reduced. Consequently, we propose that the ciliary genes identified in this study, especially *Tctex1d2* and *Cfap45*, play crucial roles in the larval settlement, and MIP2 promotes *U. unicincus* larval settlement by regulating the expression of these ciliary genes.

**Table 3:** Functions and literature sources of the differential expressed ciliary genes in the larval transcriptomes between the MIP2 treatment and control groups

Gene name	Functions	Literature sources
*Tctex1d2* (Tctex1 domain-containing protein 2)	Intraflagellar transport	[96–99]
*Cav1* (Caveolin-1)	Control ciliary membrane composition	[82–84]
*β-tubulin*	Composed of eukaryotic ciliary microtubules	[100, 101]
*Cfap45* (Cilia- and flagella-associated protein 45)	Sustain cilia stability	[60, 79]
*α-tubulin*	Composed of eukaryotic ciliary microtubules	[101, 102]
*Bbs12* (Bardet-Biedl syndrome 12)	Involved in vesicle trafficking of ciliary membrane	[95]
*Ift43* (Intraflagellar transport protein 43)	Intraflagellar transport	[85–87]
*Mns1* (Meiosis-specific nuclear structural protein 1)	Involved in ciliogenesis and ciliary beating	[80, 81]
*Ttc25* (Tetratricopeptide repeat protein 25)	Intraflagellar transport	[104]
*Anapc2* (Anaphase-promoting complex subunit 2)	Intraflagellar transport	[94]
*Wdpcp* (WD repeat-containing and planar cell polarity effector protein fritz homolog)	Intraflagellar transport	[102, 103]
*Ift22* (Intraflagellar transport protein 22)	Intraflagellar transport	[91–93]
*Wdr60* (WD repeat-containing protein 60)	Intraflagellar transport	[96, 97, 99]
*Ift74* (Intraflagellar transport protein 74)	Intraflagellar transport	[88–90]

## Conclusions

Our study establishes that MIP2 serves as the principal neuropeptide inducing the larval settlement process in *U. unicinctus* by the SPR-cAMP-PKA pathway, thereby regulating the expression of pivotal downstream ciliary genes, including the key gene *Tctex1d2*. Notably, our investigation highlights the substantial involvement of energy metabolism and signal transduction pathways in the MIP2-induced larval settlement of *U. unicinctus*. To our knowledge, this research represents the inaugural documentation of the action pathway through which the neuropeptide MIP induces the larval settlement in *U. unicinctus*. The insights garnered from our findings are poised to significantly contribute to future research endeavors focused on unraveling the intricacies of neuropeptide regulation in larval settlement process.

### Electronic supplementary material

Below is the link to the electronic supplementary material.

Supplementary Material 1

Supplementary Material 2

Supplementary Material 3

Supplementary Material 4

Supplementary Material 5

Supplementary Material 6

Supplementary Material 7

Supplementary Material 8

Supplementary Material 9

Supplementary Material 10

Supplementary Material 11

Supplementary Material 12

Supplementary Material 13

Supplementary Material 14

Supplementary Material 15

Supplementary Material 16

Supplementary Material 17

Supplementary Material 18

## Data Availability

The datasets analyzed during the current study are available in the NCBI SRA repository (PRJNA485379 and PRJNA1027755).

## Abbreviations

MIP: Myoinhibitory peptide
SPR: Sex peptide receptor
MGIC: myoinhibitory peptide-gated ion channel
RT-qPCR: Real-time quantitative PCR
WISH: Whole-mount in situ hybridization
FSW: Filtered seawater
RH: Relative height
PKA: Protein kinase A
CRE: cAMP-response elements
DEGs: Differentially expressed genes
*EGFP*: Enhanced green fluorescent protein gene
RNAi: RNA interference
GO: Gene Ontology
KEGG: Kyoto Encyclopedia of Genes and Genomes
MAG: Myoinhibitory peptide activated GPCR
ADCY3: Adenylate cyclase 3
PDE4: Phosphodiesterase 4
ECM: Extracellular matrix
TPM: Transcripts per million
EC: Early cells
MC: Multicellular embryo
BL: Blastula
GA: Gastrula
ET: Early-trochophore
MT: Mid-trochophore
LT: Late-trochophore
ES: Early-segmentation larva
SL: Segmentation larva
WL: Worm-shaped larva
*Cfap45*: Cilia- and flagella-associated protein 45
*Mns1*: Meiosis-specific nuclear structural protein 1
*Cav1*: Caveolin-1
*Ift43*: Intraflagellar transport protein 43
*Ift74*: Intraflagellar transport protein 74
*Ift22*: Intraflagellar transport protein 22
*Tctex1d2*: Tctex1 domain-containing protein 2

## References

[1] Toonen RJ, Pawlik JR (1994). Foundations of gregariousness. Nature.

[2] Qian PY (1999). Larval settlement of polychaetes. Hydrobiologia.

[3] Hadfield MG, Carpizo-Ituarte EG, Carmen KD, Nedved BT (2001). Metamorphic competence, a major adaptive convergence in marine invertebrate larvae. Am Zool.

[4] Marshall DJ, Krug PJ, Kupriyanova EK, Byrne M, Emlet RB (2013). The biogeography of marine invertebrate life histories. Annu Rev Ecol Evol S.

[5] Shikuma NJ, Pilhofer M, Weiss GL, Hadfield MG, Jensen GJ, Newman DK (2014). Marine tubeworm metamorphosis induced by arrays of bacterial phage tail-like structures. Science.

[6] Rodriguez SR, Ojeda FP, Inestrosa NC (1993). Settlement of benthic marine-invertebrates. Mar Ecol Prog Ser.

[7] Dreanno C, Matsumura K, Dohmae N, Takio K, Hirota H, Kirby RR (2006). An alpha(2)-macroglobulin-like protein is the cue to gregarious settlement of the barnacle *Balanus amphitrite*. Proc Natl Acad Sci U S A.

[8] Grant MN, Meritt DW, Kimmel DG (2013). Chemical induction of settlement behavior in larvae of the eastern oyster *Crassostrea virginica* (Gmelin). Aquaculture.

[9] Sneed JM, Sharp KH, Ritchie KB, Paul VJ (2014). The chemical cue tetrabromopyrrole from a biofilm bacterium induces settlement of multiple Caribbean corals. Proc R Soc B.

[10] Moeller M, Nietzer S, Schupp PJ (2019). Neuroactive compounds induce larval settlement in the scleractinian coral *Leptastrea purpurea*. Sci Rep.

[11] Schmich J, Trepel S, Leitz T (1998). The role of GLWamides in metamorphosis of *Hydractinia Echinate*. Dev Genes Evol.

[12] Erwin PM, Szmant AM (2010). Settlement induction of *Acropora palmata* planulae by a GLW-amide neuropeptide. Coral Reefs.

[13] Grasso LC, Negri AP, Foret S, Saint R, Hayward DC, Miller DJ (2011). The biology of coral metamorphosis: molecular responses of larvae to inducers of settlement and metamorphosis. Dev Biol.

[14] Whalan S, Webster NS, Negri AP (2012). Crustose coralline algae and a cnidarian neuropeptide trigger larval settlement in two coral reef sponges. PLoS ONE.

[15] Williams EA (2020). Function and distribution of the Wamide neuropeptide superfamily in metazoans. Front Endocrinol.

[16] Conzelmann M, Williams EA, Tunaru S, Randel N, Shahidi R, Asadulina A (2013). Conserved MIP receptor-ligand pair regulates *Platynereis* larval settlement. Proc Natl Acad Sci U S A.

[17] Yan GY, Zhang G, Huang JM, Lan Y, Sun J, Zeng C (2017). Comparative transcriptomic analysis reveals candidate genes and pathways involved in larval settlement of the barnacle *Megabalanus volcano*. Int J Mol Sci.

[18] Schoofs L, Holman GM, Hayes TK, Nachman RJ, Deloof A (1991). Isolation, identification and synthesis of locustamyoinhibiting peptide (lom-mip), a novel biologically-active neuropeptide from *Locusta Migratoria*. Regul Pept.

[19] Hua YJ, Tanaka Y, Nakamura K, Sakakibara M, Nagata S, Kataoka H (1999). Identification of a prothoracicostatic peptide in the larval brain of the silkworm, *Bombyx mori*. J Biol Chem.

[20] Davis NT, Blackburn MB, Golubeva EG, Hildebrand JG (2003). Localization of myoinhibitory peptide immunoreactivity in *Manduca sexta* and *Bombyx mori*, with indications that the peptide has a role in molting and ecdysis. J Exp Biol.

[21] Santos JG, Vomel M, Struck R, Homberg U, Nassel DR, Wegener C (2007). Neuroarchitecture of peptidergic systems in the larval ventral ganglion of *Drosophila melanogaster*. PLoS ONE.

[22] Rewitz KF, Yamanaka N, Gilbert LI, O’Connor MB (2009). The insect neuropeptide ptth activates receptor tyrosine kinase torso to initiate metamorphosis. Science.

[23] Yamanaka N, Hua YJ, Roller L, Spalovska-Valachova I, Mizoguchi A, Kataoka H (2010). *Bombyx* prothoracicostatic peptides activate the sex peptide receptor to regulate ecdysteroid biosynthesis. Proc Natl Acad Sci U S A.

[24] Szabo TM, Chen RB, Goeritz ML, Maloney RT, Tang LS, Li LJ (2011). Distribution and physiological effects of B-type allatostatins (myoinhibitory peptides, MIPs) in the stomatogastric nervous system of the crab *Cancer borealis*. J Comp Neurol.

[25] Williams EA, Conzelmann M, Jekely G (2015). Myoinhibitory peptide regulates feeding in the marine annelid *Platynereis*. Front Zool.

[26] Yapici N, Kim YJ, Ribeiro C, Dickson BJ (2008). A receptor that mediates the post-mating switch in *Drosophila* reproductive behaviour. Nature.

[27] Kim YJ, Bartalska K, Audsley N, Yamanaka N, Yapici N, Lee JY (2010). MIPs are ancestral ligands for the sex peptide receptor. Proc Natl Acad Sci U S A.

[28] Oh Y, Yoon SE, Zhang Q, Chae HS, Daubnerova I, Shafer OT (2014). A homeostatic sleep-stabilizing pathway in *Drosophila* composed of the sex peptide receptor and its ligand, the myoinhibitory peptide. PLoS Biol.

[29] Peymen K, Watteyne J, Borghgraef C, Van Sinay E, Beets I, Schoofs L (2019). Myoinhibitory peptide signaling modulates aversive gustatory learning in *Caenorhabditis elegans*. PLoS Genet.

[30] Schmidt A, Bauknecht P, Williams EA, Augustinowski K, Grunder S, Jekely G (2018). Dual signaling of Wamide myoinhibitory peptides through a peptide-gated channel and a GPCR in *Platynereis*. FASEB J.

[31] Zheng QJ, Wang YJ, Chen J, Li YP, Zhao F, Liu DW (2022). Effects of salinity on the growth, physiological characteristics, and intestinal microbiota of the Echiura worm (*Urechis unicinctus*). Front Mar Sci.

[32] Chen W, Zhang S, Sun Y, Tian B, Song L, Xu Y (2021). Effects of substrate on the physiological characteristics and intestinal microbiota of Echiura worm (*Urechis unicinctus*) juveniles. Aquaculture.

[33] Tang YZ, Ma S, Liu YH, Pi YR, Liu Y, Zhao Y (2021). Intestinal microbial diversity and functional analysis of *Urechis unicinctus* from two different habitats: pond polycultured with *Penaeus japonicus* and coastal zone. Aquacult Env Interac.

[34] Hou XT, Qin ZK, Wei MK, Fu Z, Liu RN, Lu L (2020). Identification of the neuropeptide precursor genes potentially involved in the larval settlement in the Echiuran worm *Urechis Unicinctus*. BMC Genomics.

[35] Lu L, Zhang ZF, Zheng QJ, Chen ZT, Bai SM, Zhang ZR (2022). Expression characteristics and potential function of neuropeptide mip in larval settlement of the Echiuran worm *Urechis Unicinctus*. J Ocean Univ China.

[36] Bai SM, Fan ST, Liu DW, Zhang ZR, Zhang ZF (2022). Identification and expression analysis of receptors that mediate MIP regulating larval settlement in *Urechis unicinctus*. Comp Biochem Phys B.

[37] Wei MK, Qin ZK, Kong DX, Liu DW, Zheng QJ, Bai SM et al. Echiuran Hox genes provide new insights into the correspondence between Hox subcluster organization and collinearity pattern. Proc R Soc B. 2022;289(1982):20220705. 10.1098/rspb.2022.0705 PMC9449475 36264643

[38] Grabherr MG, Haas BG, Yassour M, Levin JZ, Thompson DA, Amit I (2011). Full-length transcriptome assembly from RNA-Seq data without a reference genome. Nat Biotechnol.

[39] Simao FA, Waterhouse RM, Ioannidis P, Kriventseva EV, Zdobnov EM (2015). BUSCO: assessing genome assembly and annotation completeness with single-copy orthologs. Bioinformatics.

[40] Luo WW, Cao XJ, Xu XW, Huang SQ, Liu CS, Tomljanovic T (2016). Developmental transcriptome analysis and identification of genes involved in formation of intestinal air-breathing function of Dojo Loach, *Misgurnus anguillicaudatus*. Sci Rep.

[41] Li B, Dewey CN (2011). RSEM: accurate transcript quantification from RNA-Seq data with or without a reference genome. BMC Bioinformatics.

[42] Wagner GP, Kin K, Lynch VJ (2012). Measurement of mRNA abundance using RNA-seq data: RPKM measure is inconsistent among samples. Theory Biosci.

[43] Love MI, Huber W, Anders S (2014). Moderated estimation of Fold change and dispersion for RNA-seq data with DESeq2. Genome Biol.

[44] Mao XZ, Cai T, Olyarchuk JG, Wei LP (2005). Automated genome annotation and pathway identification using the KEGG Orthology (KO) as a controlled vocabulary. Bioinformatics.

[45] Dadová P, Mikulová A, Jarous R, Chorvátová M, Uldrijan S, Kubala L (2023). A forskolin-mediated increase in cAMP promotes T helper cell differentiation into the Th1 and Th2 subsets rather than into the Th17 subset. Int Immunopharmacol.

[46] Wei M, Lu L, Wang Q, Kong D, Zhang T, Qin Z (2019). Evaluation of suitable reference genes for normalization of RT-qPCR in Echiura (*Urechis unicinctus*) during developmental process. Russ J Mar Biol.

[47] Conzelmann M, Offenburger SL, Asadulina A, Keller T, Munch TA, Jekely G (2011). Neuropeptides regulate swimming depth of *Platynereis* larvae. Proc Natl Acad Sci U S A.

[48] Wang YQ, Liu Q, Zhou Y, Chen LZ, Yang YM, Shi X (2023). Stage-specific transcriptomes of the mussel *Mytilus coruscus* reveals the developmental program for the planktonic to benthic transition. Genes.

[49] Li C, Kim K, Neuropeptides. WormBook: Online Rev C Elegans Biology. 2008:1–36. 10.1895/wormbook.1.142.1 PMC2749236 18819171

[50] Lubawy J, Marciniak P, Rosinski G, Identification (2020). Localization in the central nervous system and novel myostimulatory effect of allatostatins in *Tenebrio molitor* beetle. Int J Mol Sci.

[51] Yang XX, Zhang Y, Wong YH, Qian PY (2018). HSP90 regulates larval settlement of the bryozoan *Bugula neritina* through the nitric oxide pathway. J Exp Biol.

[52] Tsuzuki M, Moskvin OV, Kuribayashi M, Sato K, Retamal S, Abo M (2011). Salt stress-induced changes in the transcriptome, compatible solutes, and membrane lipids in the facultatively phototrophic bacterium *Rhodobacter sphaeroides*. Appl Environ Microb.

[53] Liew KJ, Zhang XH, Cai XH, Ren DD, Liu W, Chang ZD et al. Transcriptome study of cold plasma treated *Pseudomonas aeruginosa*. Chiang Mai J Sci. 2023;50(2).

[54] Junttila S, Laiho A, Gyenesei A, Rudd S (2013). Whole transcriptome characterization of the effects of dehydration and rehydration on *Cladonia Rangiferina*, the grey reindeer lichen. BMC Genomics.

[55] Barroso L (2018). ADCY3, neuronal primary cilia and obesity. Nat Genet.

[56] Vecsey CG, Baillie GS, Jaganath D, Havekes R, Daniels A, Wimmer M (2009). Sleep deprivation impairs cAMP signalling in the hippocampus. Nature.

[57] Maier LS, Bers DM (2007). Role of Ca^2+^/calmodulin-dependent protein kinase (CaMK) in excitation-contraction coupling in the heart. Cardiovasc Res.

[58] Kim JK, Choi JW, Lim S, Kwon O, Seo JK, Ryu SH (2011). Phospholipase C-η1 is activated by intracellular Ca^2+^ mobilization and enhances GPCRs/PLC/Ca^2+^ signaling. Cell Signal.

[59] Ravi P, Trivedi D, Hasan G (2018). FMRFa receptor stimulated Ca^2+^ signals alter the activity of flight modulating central dopaminergic neurons in *Drosophila melanogaster*. PLoS Genet.

[60] Dougherty GW, Mizuno K, Nothe-Menchen T, Ikawa Y, Boldt K, Ta-Shma A (2020). CFAP45 deficiency causes situs abnormalities and asthenospermia by disrupting an axonemal adenine nucleotide homeostasis module. Nat Commun.

[61] Zhang Y, Xu Y, Arellano SM, Xiao K, Qian PY (2010). Comparative proteome and phosphoproteome analyses during cyprid development of the barnacle *Balanus* (= *Amphibalanus*) *Amphitrite*. J Proteome Res.

[62] Chen ZF, Zhang HM, Wang H, Matsumura K, Wong YH, Ravasi T (2014). Quantitative proteomics study of larval settlement in the barnacle *Balanus amphitrite*. PLoS ONE.

[63] Zhang G, Yan GY, Yang XX, Wong YH, Sun J, Zhang Y (2016). Characterization of arginine kinase in the barnacle *Amphibalanus Amphitrite* and its role in the larval settlement. J Exp Zool Part B.

[64] Jiang ZX, Ping SS, Jin CL, Tu CD, Zhou XJ (2022). Transcriptome analysis provides insights into a molecular mechanism of histamine response in the cyprid larvae of *Amphibalanus amphitrite*. Mar Ecol Prog Ser.

[65] Di GL, Xiao XH, Tong MH, Chen XH, Li L, Huang MQ, et al. Proteome of larval metamorphosis induced by epinephrine in the Fujian oyster *Crassostrea Angulate*. BMC Genomics. 2020;21(1):675. 10.1186/s12864-020-07066-z PMC7525975 32993483

[66] Chandramouli KH, Sun J, Mok FSY, Liu L, Qiu JW, Ravasi T (2013). Transcriptome and quantitative proteome analysis reveals molecular processes associated with larval metamorphosis in the Polychaete *Pseudopolydora Vexillosa*. J Proteome Res.

[67] Shikuma NJ, Antoshechkin I, Medeiros JM, Pilhofer M, Newman DK (2016). Stepwise metamorphosis of the tubeworm *Hydroides elegans* is mediated by a bacterial inducer and MAPK signaling. Proc Natl Acad Sci U S A.

[68] Cao FJ, Zhong RZ, Yang CY, Hao RJ, Wang QH, Liao YS (2020). Transcriptomic analysis of differentially expressed genes in the larval settlement and metamorphosis of peanut worm *Sipunculus Nudus*. Aquacult Rep.

[69] He J, Wu ZW, Chen LY, Dai Q, Hao HH, Su P (2021). Adenosine Triggers larval settlement and metamorphosis in the mussel *Mytilopsis sallei* through the ADK-AMPK-FoxO pathway. ACS Chem Biol.

[70] Hardie DG (2011). AMP-activated protein kinase-an energy sensor that regulates all aspects of cell function. Genes Dev.

[71] Marinkovic M, Berger J, Jekely G (2020). Neuronal coordination of motile cilia in locomotion and feeding. Philos Trans R Soc B.

[72] Kong DX, Wei MK, Liu DW, Zhang ZR, Ma YB, Zhang ZF (2023). Morphological observation and transcriptome analysis of ciliogenesis in *Urechis unicinctus* (Annelida, Echiura). Int J Mol Sci.

[73] Mackie GO, Paul DH, Singla CM, Sleigh MA, Williams DE (1974). Branchial innervation and ciliary control in the ascidian *Corella*. Proc R Soc Lond Ser B.

[74] Arkett SA (1987). Ciliary arrest controlled by identified central neurons in a urochordate (ascidiacea). J Comp Physiol A.

[75] Veraszto C, Ueda N, Bezares-Calderon LA, Panzera A, Williams EA, Shahidi R (2017). Ciliomotor circuitry underlying whole-body coordination of ciliary activity in the *Platynereis* larva. eLife.

[76] Penniman JR, Doll MK, Pires A (2013). Neural correlates of settlement in veliger larvae of the gastropod, *Crepidula Fornicate*. Invertebr Biol.

[77] Thiel D, Bauknecht P, Jekely G, Hejnol A (2017). An ancient FMRFamide-related peptide-receptor pair induces defence behaviour in a brachiopod larva. Open Biol.

[78] Thiel D, Bauknecht P, Jekely G, Hejnol A (2019). A nemertean excitatory peptide/CCHamide regulates ciliary swimming in the larvae of *Lineus longissimus*. Front Zool.

[79] Deniz E, Pasha M, Guerra ME, Viviano S, Ji M, Konstantino M (2023). CFAP45, a heterotaxy and congenital heart disease gene, affects cilia stability. Dev Biol.

[80] Zhou J, Yang F, Leu NA, Wang PJ (2012). MNS1 is essential for spermiogenesis and motile ciliary functions in mice. PLoS Genet.

[81] Ta-Shma A, Hjeij R, Perles Z, Dougherty GW, Abu Zahira I, Letteboer SJF (2018). Homozygous loss-of-function mutations in MNS1 cause laterality defects and likely male infertility. PLoS Genet.

[82] Arenas-Mena C, Wong KSY, Arandi-Forosani N (2007). Ciliary band gene expression patterns in the embryo and trochophore larva of an indirectly developing polychaete. Gene Expr Patterns.

[83] Scheidel N, Kennedy J, Blacque OE (2018). Endosome maturation factors Rabenosyn-5/VPS45 and caveolin-1 regulate ciliary membrane and polycystin-2 homeostasis. EMBO J.

[84] Wang J, Zhang LL, Lian SS, Qin ZK, Zhu X, Dai XT (2020). Evolutionary transcriptomics of metazoan biphasic life cycle supports a single intercalation origin of metazoan larvae. Nat Ecol Evol.

[85] Behal RH, Miller MS, Qin HM, Lucker BF, Jones A, Cole DG (2012). Subunit interactions and organization of the *Chlamydomonas reinhardtii* intraflagellar transport complex A proteins. J Biol Chem.

[86] Biswas P, Duncan JL, Ali M, Matsui H, Naeem MA, Raghavendra PB (2017). A mutation in IFT43 causes non-syndromic recessive retinal degeneration. Hum Mol Genet.

[87] Zhu B, Zhu X, Wang LM, Liang YM, Feng QQ, Pan JM (2017). Functional exploration of the IFT-A complex in intraflagellar transport and ciliogenesis. PLoS Genet.

[88] Bhogaraju S, Cajanek L, Fort C, Blisnick T, Weber K, Taschner M (2013). Molecular basis of tubulin transport within the cilium by IFT74 and IFT81. Science.

[89] Brown JM, Cochran DA, Craige B, Kubo T, Witman GB (2015). Assembly of IFT trains at the ciliary base depends on IFT74. Curr Biol.

[90] Zhu PP, Xu JJ, Wang YD, Zhao CT (2021). Loss of Ift74 leads to slow photoreceptor degeneration and ciliogenesis defects in zebrafish. Int J Mol Sci.

[91] Mei X, Westfall TA, Zhang QH, Sheffield VC, Bassuk AG, Slusarski DC (2014). Functional characterization of Prickle2 and BBS7 identify overlapping phenotypes yet distinct mechanisms. Dev Biol.

[92] Wachter S, Jung JM, Shafiq S, Basquin J, Fort C, Bastin P (2019). Binding of IFT22 to the intraflagellar transport complex is essential for flagellum assembly. EMBO J.

[93] Xue B, Liu YX, Dong B, Wingfield JL, Wu MF, Sun J (2020). Intraflagellar transport protein RABL5/IFT22 recruits the BBSome to the basal body through the GTPase ARL6/BBS3. Proc Natl Acad Sci U S A.

[94] Ganner A, Lienkamp S, Schafer T, Romaker D, Wegierski T, Park TJ (2009). Regulation of ciliary polarity by the APC/C. Proc Natl Acad Sci U S A.

[95] Seo S, Baye LM, Schulz NP, Beck JS, Zhang QH, Slusarski DC (2010). BBS6, BBS10, and BBS12 form a complex with CCT/TRiC family chaperonins and mediate BBSome assembly. Proc Natl Acad Sci U S A.

[96] Asante D, Stevenson NL, Stephens DJ (2014). Subunit composition of the human cytoplasmic dynein-2 complex. J Cell Sci.

[97] Gholkar AA, Senese S, Lo YC, Capri J, Deardorff WJ, Dharmarajan H (2015). Tctex1d2 associates with short-rib polydactyly syndrome proteins and is required for ciliogenesis. Cell Cycle.

[98] Schmidts M, Hou YQ, Cortes CR, Mans DA, Huber C, Boldt K (2015). TCTEX1D2 mutations underlie Jeune asphyxiating thoracic dystrophy with impaired retrograde intraflagellar transport. Nat Commun.

[99] Hamada Y, Tsurumi Y, Nozaki S, Katoh Y, Nakayama K (2018). Interaction of WDR60 intermediate chain with TCTEX1D2 light chain of the dynein-2 complex is crucial for ciliary protein trafficking. Mol Biol Cell.

[100] Dutcher SK (2001). The tubulin fraternity: alpha to eta. Curr Opin Cell Biol.

[101] Pathak N, Obara T, Mangos S, Liu Y, Drummond IA (2007). The zebrafish fleer gene encodes an essential regulator of cilia tubulin polyglutamylation. Mol Biol Cell.

[102] Toriyama M, Lee CJ, Taylor SP, Duran I, Cohn DH, Bruel AL (2016). The ciliopathy-associated CPLANE proteins direct basal body recruitment of intraflagellar transport machinery. Nat Genet.

[103] Ma Y, Tian P, Zhong H, Wu F, Zhang QN, Liu X (2021). WDPCP modulates cilia beating through the mapk/erk pathway in chronic rhinosinusitis with nasal polyps. Front Cell Dev Biol.

[104] Wallmeier J, Shiratori H, Dougherty GW, Edelbusch C, Hjeij R, Loges NT (2016). TTC25 deficiency results in defects of the outer dynein arm docking machinery and primary ciliary dyskinesia with left-right body asymmetry randomization. Am J Hum Genet.

