# Supplementary figures and images for: Identification of the principal neuropeptide MIP and its action pathway in larval settlement of the echiuran worm Urechis unicinctus
Source: BMC Genomics. 2024 Apr 3;25:337. doi: 10.1186/s12864-024-10228-y (PMC11027379; doi:10.1186/s12864-024-10228-y)

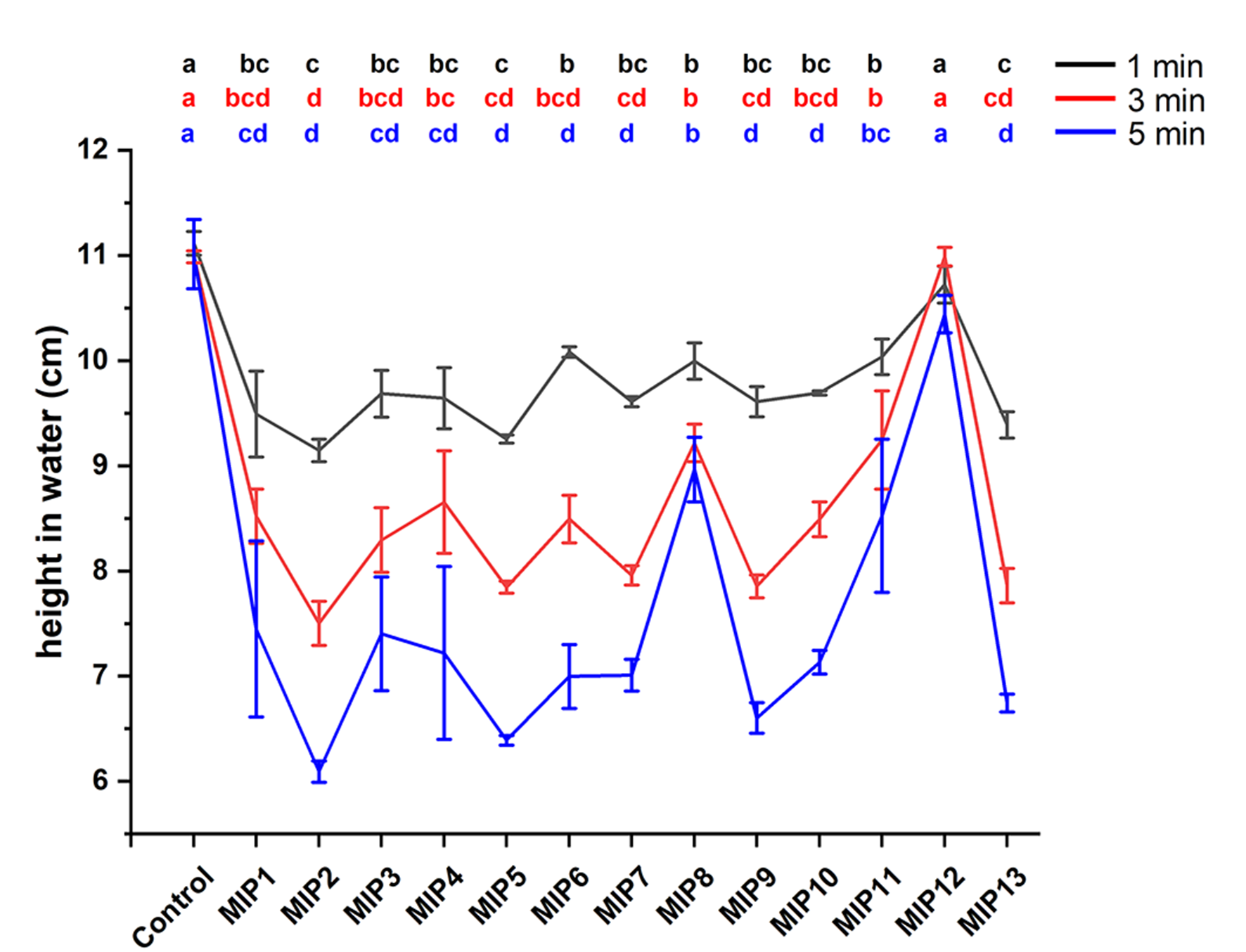

Supplement: Supplementary file 7 — Supplementary Material 7 [file 12864_2024_10228_MOESM7_ESM.png]

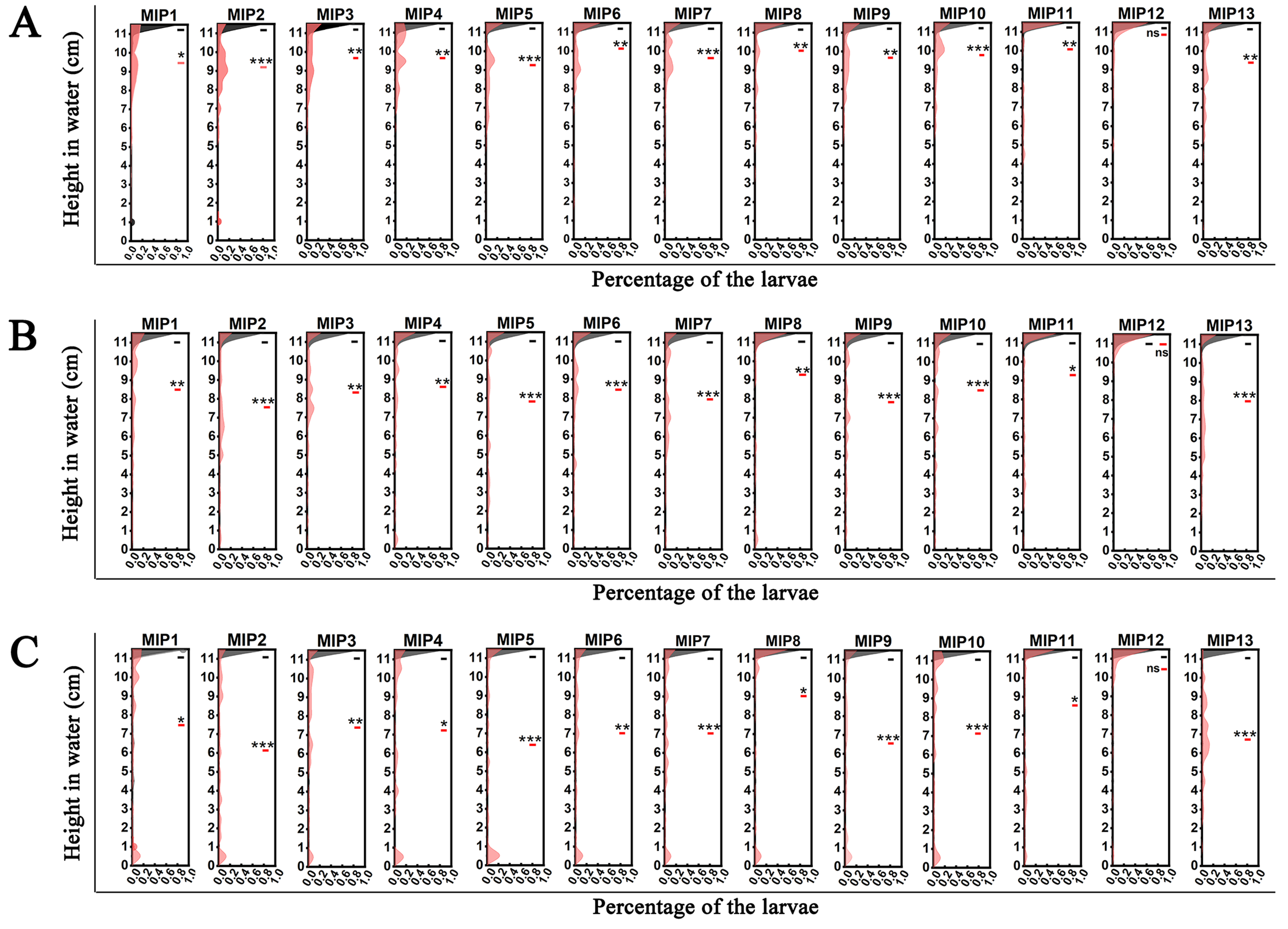

Supplement: Supplementary file 8 — Supplementary Material 8 [file 12864_2024_10228_MOESM8_ESM.png]

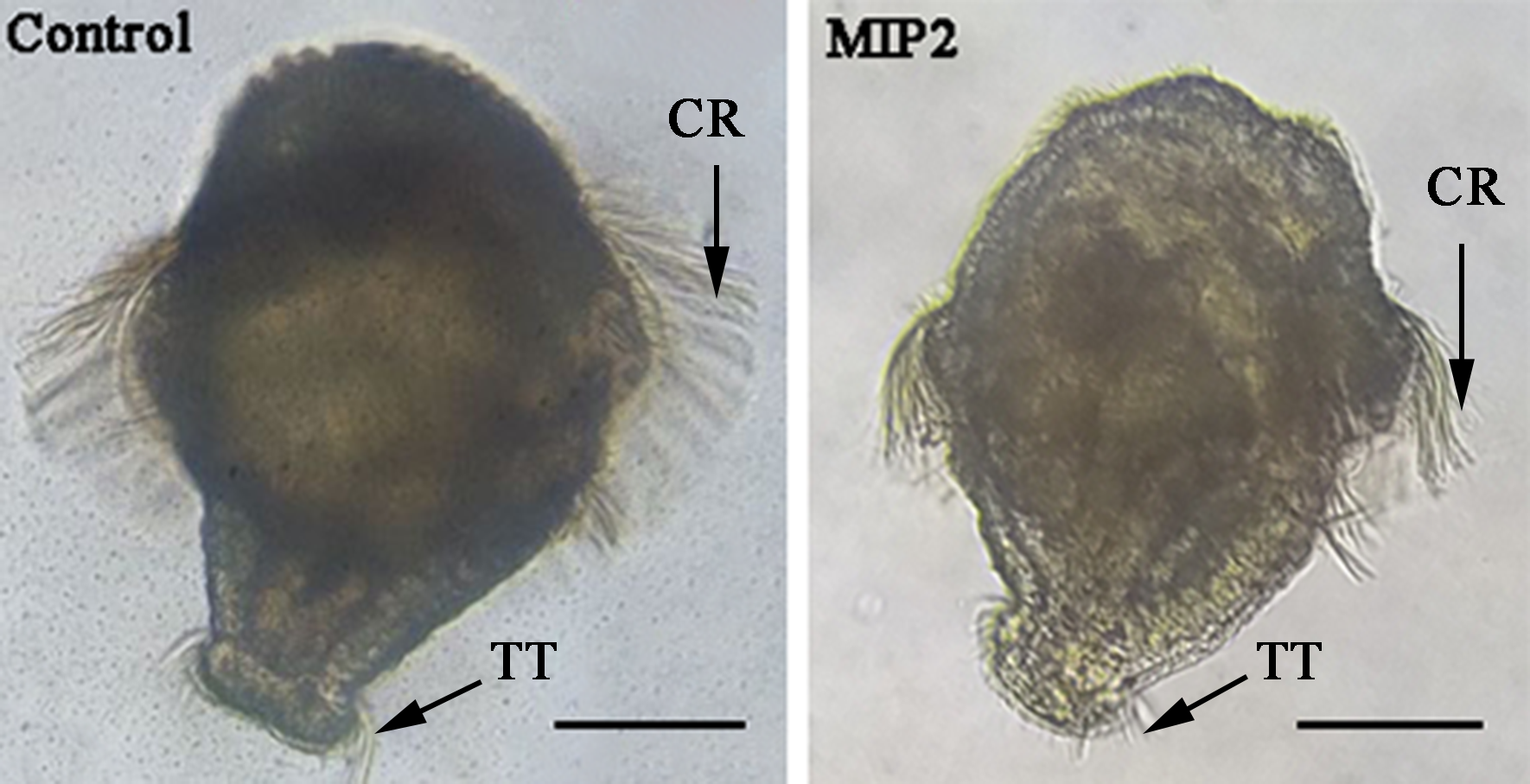

Supplement: Supplementary file 9 — Supplementary Material 9 [file 12864_2024_10228_MOESM9_ESM.png]

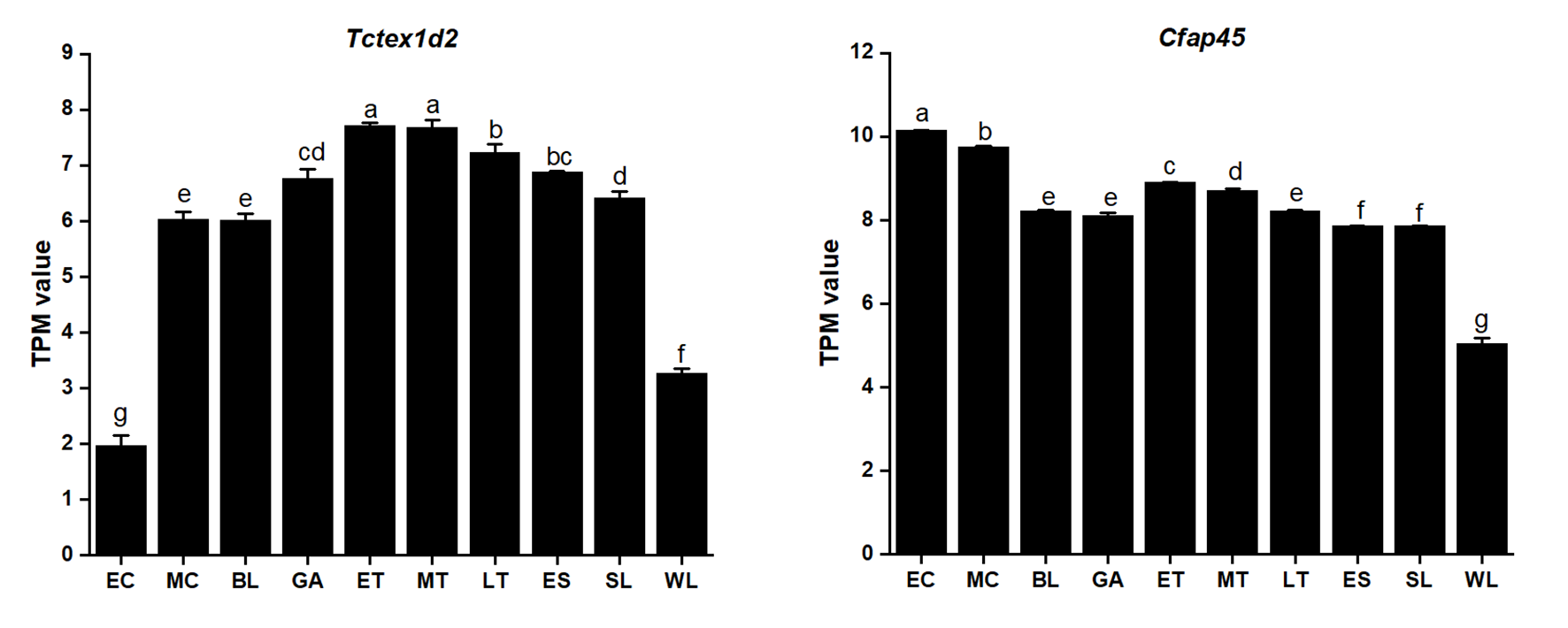

Supplement: Supplementary file 14 — Supplementary Material 14 [file 12864_2024_10228_MOESM14_ESM.png]

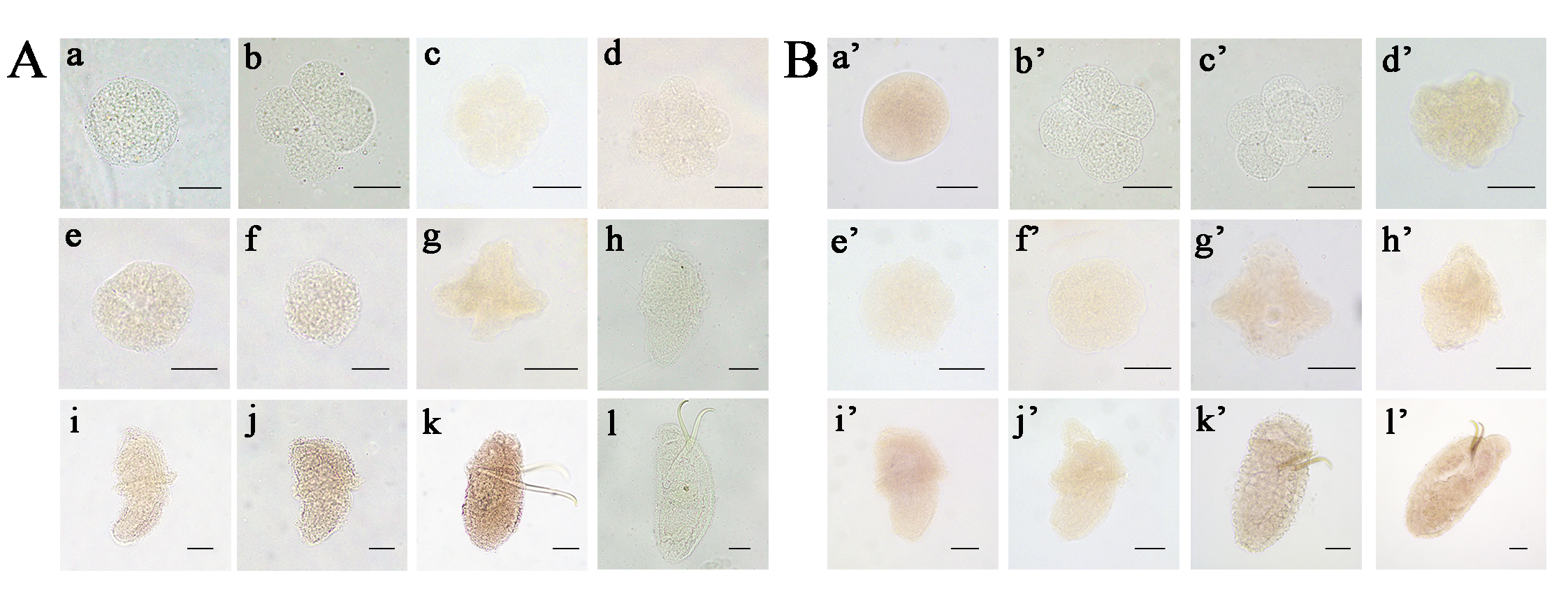

Supplement: Supplementary file 15 — Supplementary Material 15 [file 12864_2024_10228_MOESM15_ESM.png]

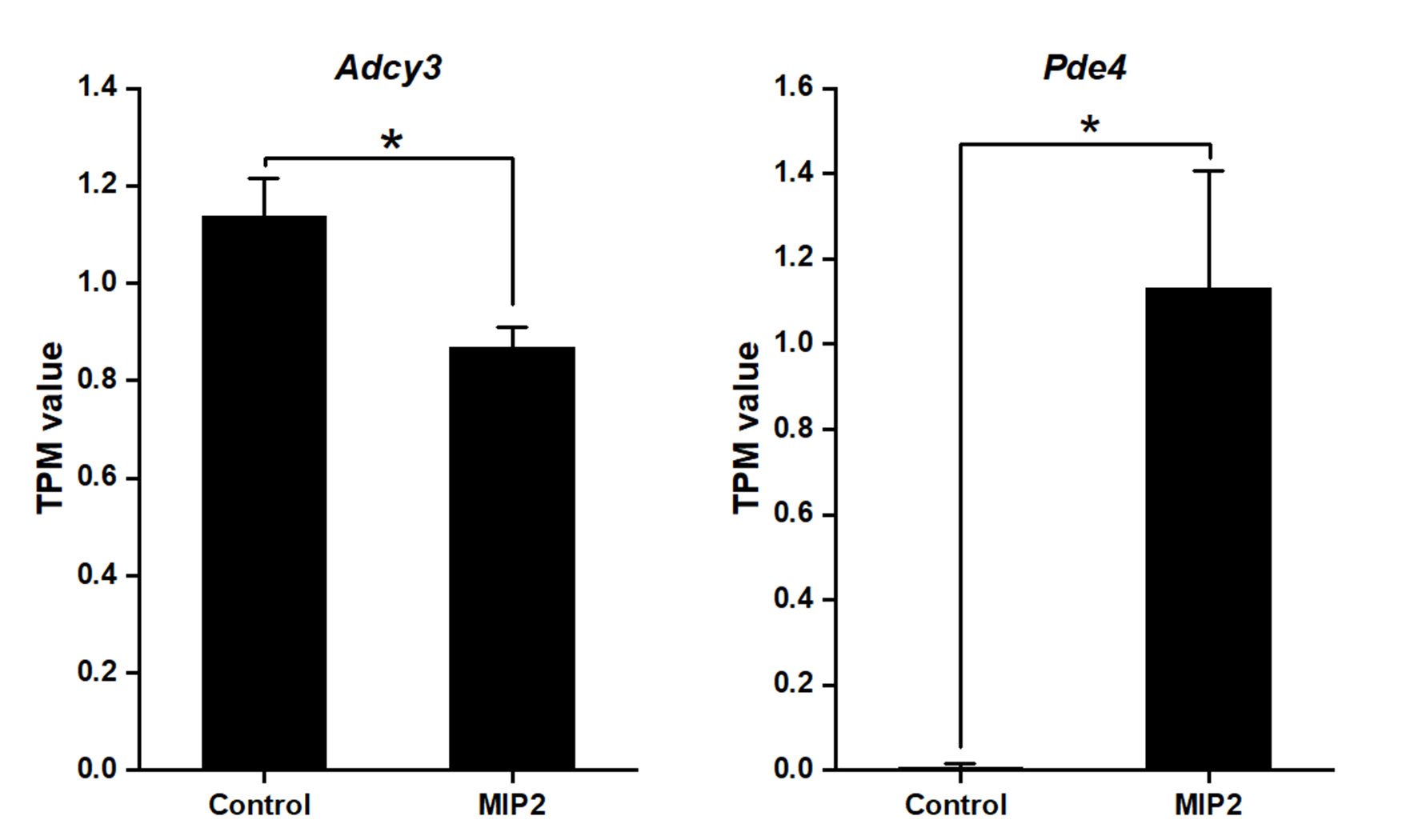

Supplement: Supplementary file 16 — Supplementary Material 16 [file 12864_2024_10228_MOESM16_ESM.png]

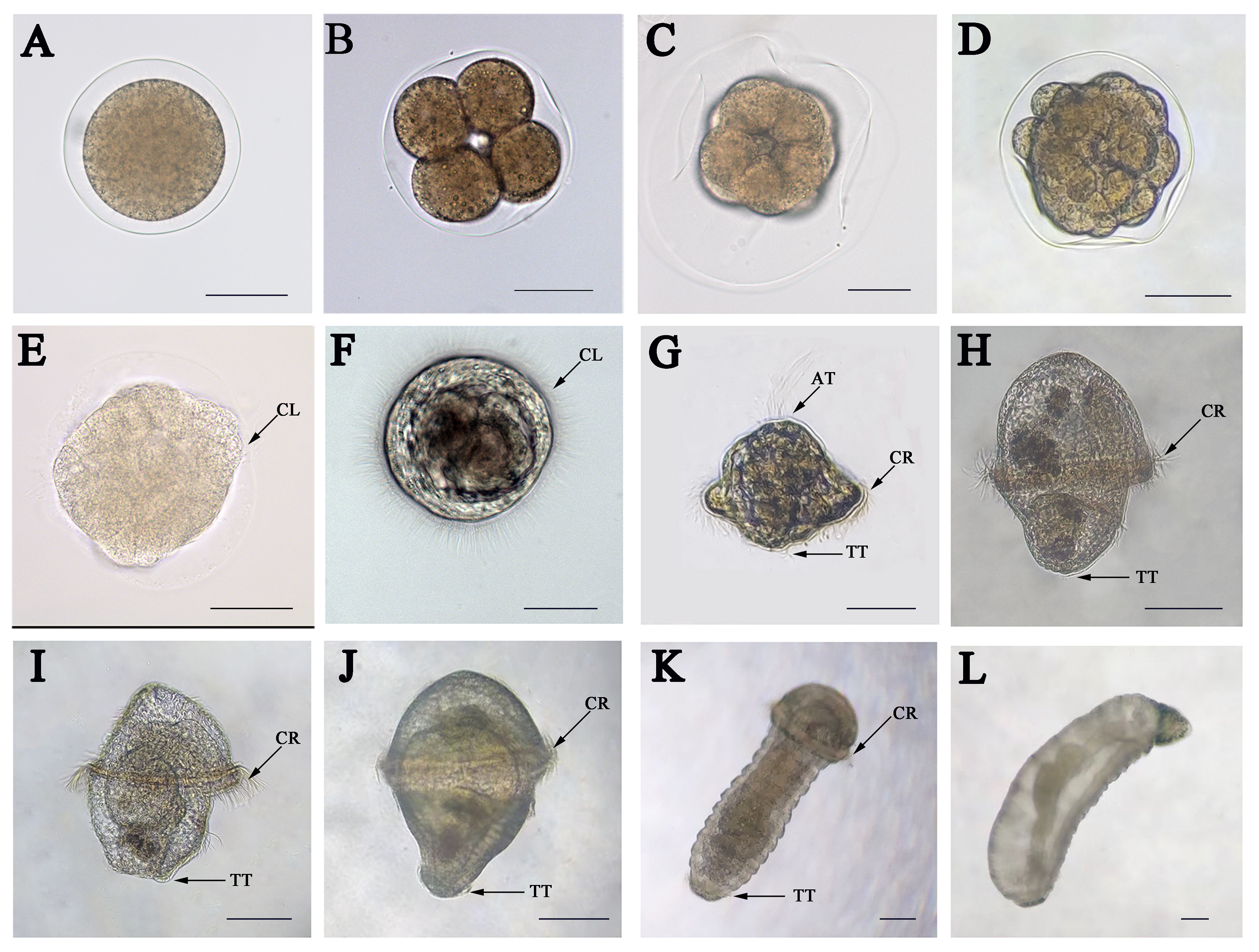

Supplement: Supplementary file 17 — Supplementary Material 17 [file 12864_2024_10228_MOESM17_ESM.png]
