# Supplementary material for: Identification of the principal neuropeptide MIP and its action pathway in larval settlement of the echiuran worm Urechis unicinctus
Source: BMC Genomics. 2024 Apr 3;25:337. doi: 10.1186/s12864-024-10228-y (PMC11027379; doi:10.1186/s12864-024-10228-y)
Supplement: Supplementary file 18 — Supplementary Material 18 [file 12864_2024_10228_MOESM18_ESM.docx]

**Additional file 1: Table S1.** Amino acid sequences of *U. unicinctus* MIP mature peptides. “a” indicates the carboxyl terminal amidation in the last amino acid (W) of MIP mature peptides.

**Additional file 2: Table S2.** Primers for *Spr*-ORF cloning.

**Additional file 3: Table S3.** Specific primers for RT-qPCR.

**Additional file 4: Table S4.** Specific primers for template amplification of the probe used in WISH.

**Additional file 5: Table S5.** Primer sequences for the synthesis of *Tctex1d2*-dsRNA and *EGFP*-dsRNA templates.

**Additional file 6：Video S1.** Settlement of *U*. *unicinctus* early-segmentation larvae treated by neuropeptide MIP2. The video is processed at 30 × acceleration.

**Additional file 7: Fig. S1.** Height of larvae in water after treated by 13 MIPs at different times. Data are presented as the mean ± SEM from three biological replicates. Different letters indicate significant differences (*p* < 0.05).

**Additional file 8: Fig. S2**. Distribution of larvae in water layer at different treatment time with MIP mature peptides. A: 1 min; B: 3 min; C: 5 min. The pink shaded area and gray shaded area represent larval distribution in the MIP treatment group and control group, respectively. The pink bar and gray bar represent the mean depth of larvae distribution in the MIP treatment group and control group. The horizontal axis shows the proportion of larvae in a certain water layer to the total number of larvae in the glass tube. Asterisks indicate significant differences between the MIP treatment groups and the control group (* *p* < 0.05; ** *p* < 0.01; *** *p* < 0.001). “ns” indicates no significant differences between the MIP treatment groups and control group.

**Additional file 9: Fig. S3.** Morphological observation of the control larvae and the early-segmentation larvae treated by MIP2 for 7 h. CR: circumoral ciliary ring; TT: telotroch. All scales are 50 μm.

**Additional file 10: Table S6.** Detailed information on genes associated with neuropeptide receptor or key signaling molecules in the larval transcriptomes.

**Additional file 11: Table S7.** Detailed information on down-regulated reported cilia-related genes in the larval transcriptomes.

**Additional file 12: Table S8.** Kyoto Encyclopedia of Genes and Genomes (KEGG) enrichment analysis of down-regulated DEGs in the larval transcriptomes.

**Additional file 13: Table S9.** Kyoto Encyclopedia of Genes and Genomes (KEGG) enrichment analysis of up-regulated DEGs in the larval transcriptomes.

**Additional file 14: Fig. S4.** mRNA abundances of *Tctex1d2* and *Cfap45* in embryos and larvae at the different developmental stages from *U. unicinctus* transcriptome (NCBI accession number: PRJNA485379). EC: 2-8 cells; MC: multicellular embryo; BL: blastula; GA: gastrula; ET: early-trochophore; MT: mid-trochophore; LT: late-trochophore; ES: early-segmentation larva; SL: segmentation larva; WL: worm-shaped larva. Data are presented as the mean ± SEM from three biological replicates. Different letters indicate significant differences (*p* < 0.05).

**Additional file 15: Fig. S5.** *U. unicinctus* embryos and larvae treated by sense probe of *Tctex1d2* and *Cfap45*. A: *Tctex1d2*; B: *Cfap45*; a and a’: zygote; b and b’: 4-cell embryo; c and c’: 8-cell embryo; d and d’: multicellular embryo; e and e’: blastula; f and f’: gastrula; g and g’: early-trochophore; h and h’: mid-trochophore; i and i’: late-trochophore; j and j’: early-segmentation larva; k and k’: late-segmentation larva; l and l’: worm-shaped larva. All scales are 50 μm.

**Additional file 16: Fig. S6.** Expression of *Adcy3* and *Pde4* in the cAMP signaling pathway (NCBI accession number: PRJNA1027755). Asterisks indicate significant differences between MIP2-treated larvae and normal early-segmentation larvae (* *p* < 0.05).

**Additional file 17: Fig. S7.** Morphological observation of cilia distribution on the surface of larvae in *U. unicinctus*. A: zygote; B: 4-cell embryo; C: 8-cell embryo; D: multicellular embryo; E: blastula; F: gastrula; G: early-trochophore; H: mid-trochophore; I: late-trochophore; J: early-segmentation larva; K: late-segmentation larva; L: worm-shaped larva. CL: surface short cilia; AT: apical ciliary tuft; CR: circumoral ciliary ring; TT: telotroch. All scales are 50 μm.
